# Targeting to high density lipoprotein cholesterol: new insights for inflammatory bowel disease treatment

**DOI:** 10.1016/j.jlr.2025.100836

**Published:** 2025-06-06

**Authors:** Xiaotong Wang, Xuefei Li, Kezhen Liu, Ke Yi, Yang Yang, Dongwen Wu, Xiaowei Liu

**Affiliations:** 1 Department of Gastroenterology, Xiangya Hospital, Central South University Changsha, Hunan, China; 2 National Clinical Research Center for Geriatric Disorders, Xiangya Hospital, Changsha, Hunan, China; 3 Department of Microbiology and Molecular Genetics, Michigan State University, East Lansing; 4 Department of Microbiology and Immunology, Cornell University, Ithaca, NY; 5 Hunan International Scientific and Technological Cooperation Base of Artificial Intelligence Computer-Aided Diagnosis and Treatment for Digestive Disease, Changsha, China; 6 Gut-Liver Axis and Intestinal Barrier Research Center, Xiangya Hospital, Central South University, Changsha, China

**Keywords:** high density lipoprotein cholesterol, inflammatory bowel disease, cholesteryl ester transfer protein inhibitors, evacetrapib, gut barrier

## Abstract

The anti-inflammatory and vasoprotective properties of HDL-C make it best known in cardiovascular disease and sepsis. We aimed to investigate whether interventions that target HDL-C metabolism may be used for the prevention and treatment of inflammatory bowel disease (IBD). The relationship between serum lipids and IBD clinical manifestations were analyzed in both respective and prospective cohort. Later, therapeutic effect and mechanism of cholesteryl ester transfer protein inhibitors (CETPis) in IBD treatment were explored by in vivo experiments. IBD patients had significantly reduced HDL-C, which was negatively correlated with their inflammatory status. Furthermore, HDL-C level was elevated by biologics agents and HDL-C concentration pretreatment was predictive for IBD patients’ future disease severity. Elevating HDL-C by CETPi before or even after the onset of experimental colitis reduced disease severity, which is associated with an ATF3-dependent anti-inflammatory reprogramming of macrophages and with enhanced gut barrier function. Together, these results demonstrate an important role of HDL-C in IBD and indicate the potential pharmacological effects of CETPi for future IBD therapy through elevation of HDL-C.

Inflammatory bowel disease (IBD), incorporating Crohn's disease (CD) and ulcerative colitis (UC), are continually increasing incidence worldwide, especially in developing nations (1). Characterized by mucosal immune dysregulation and impaired gut barrier function, IBD patients usually suffer from inappropriate systemic inflammation and cytokines storm. Chronic inflammation and inflammatory cytokines impair lipoprotein metabolism and dyslipidemia has been confirmed in diseases like systemic lupus erythematosus (2). However, a review of the literature reveals contradictory results for dyslipidemia among IBD patients (3, 4, 5, 6, 7, 8, 9, 10, 11, 12, 13, 14, 15, 16, 17, 18, 19, 20, 21, 22, 23, 24, 25, 26). Furthermore, it remains unknown whether dyslipidemia in IBD patients correlates with and plays any role in disease severity.

Recurring inflammation, as occurs in IBD, not only induces severe complications in the gut but also increases the risk of other diseases including CVD, for which dyslipidemia is considered an important risk factor (27). The role of dyslipidemia is most well-established in primary CVD, and epidemiologic studies have shown inverse associations between HDL-C levels and cardiovascular outcomes (28). As a result, cholesteryl ester transfer protein inhibitors (CETPis) targeting the major enzymatic regulator of plasma HDL-C levels were developed, including torcetrapib, dalcetrapib, anacetrapib, and evacetrapib (recently obicetrapib) (29). Disappointingly, those drugs have not been shown to reduce cardiovascular risk in clinical trials despite increasing HDL-C level (30, 31, 32, 33). HDL-C also declines drastically and acts as an early independent predictive marker of survival in severe sepsis (34). Clinical cohorts and animal in vivo experiments all demonstrated preserving HDL-C levels decreased mortality in sepsis (35) and its anti-inflammatory property via macrophage reprogramming may contribute (36). Some clinical studies have preliminarily suggested lower HDL-C levels in IBD patients (37, 38, 39), and significantly increased after corticosteroids, anti-TNFα or tofacitinib treatment (40), and high HDL-C is positively associated with mucosal healing (41). Thus, it might be worthwhile to investigate whether intervention that targets HDL-C metabolism could be used for the treatment of IBD.

Here, we hypothesized that hypolipidemia among IBD patients is related to their inflammatory state, and we questioned whether therapies elevating HDL-C could serve as new insights for IBD treatment. First, we tested this hypothesis in both retrospective and prospective clinical IBD cohort. Later, the preventive and therapeutic effects of CETPi on experimental colitis were studied. Our results comprehensively describe the clinical significance of HDL-C working as a new IBD inflammation status biomarker and reveal the capacity of improving colitis outcome by elevating HDL-C.

## Materials and methods

### Experimental design

#### Patients

Retrospectively consecutive patients who admitted to Xiangya Hospital, Central South University from January 2021 to July 2022 and were diagnosed following diseases first time were included: CD (94), UC (79), 69 healthy controls (HC, 41 for CD and 28 for UC). In addition, a prospective cohort of IBD patients from June 1, 2024, to August 1, 2024, was established to validate the retrospective cohort results. The inclusion criteria were age between 18 and 60 years, no anti-inflammatory medicine and lipid regulating agent history in past 3 months, no autoimmune diseases, inflammations, or infections at that time and in the month prior to the study; Clinical data were complete with no missing data. Patients with familiar dyslipidemia were excluded from the study. For enrolled patients, serum lipid profile, C-reactive protein (CRP), erythrocyte sedimentation rate (ESR), fibrinogen, white blood count (WBC), and serum proinflammatory cytokines (such as TNFα, interleukin (IL)-1β and IL-6) were collected at admission and during follow-up. CDAI was evaluated by gastroenterologist. Serum total protein, albumin, and body mass index (BMI) were collected to assess nutritional status. All the studies followed the Declaration of Helsinki principles.

#### Lectures systematic review and meta-analysis

PubMed, EMBASE, the Cochrane Library of Systematic Reviews, Web of Science, China National Knowledge Infrastructure, WanFang databases, and SinoMed databases were searched by using different combinations of free text and database specific index terms related to the topics. The studies were not restricted by date, language, or publication status. The following combined search term was used: (IBD, CD, UC) and (serum lipid profile, lipid panel, plasma lipid). Meta-analyses were conducted with the Stata 14.0. Studies were pooled within outcome measures, and standardized mean difference, and 95% confidence intervals were constructed using random-effects meta-analysis. Sensitive analysis was also performed to evaluate the influences of individual studies on the final effect (“leave-one out”) and the *Egger* regression asymmetry test were used to examine publication bias (42).

#### Mice

C57BL/6 mice were obtained from the Hunan SJA Laboratory Animal Co., Ltd (Changsha, China). Cholesteryl ester transfer protein (CETP transgenetic mice were gifted from National Human Disease Animal Model Resource Center (Beijing, China). IL-10 knockout mice were obtained from Shanghai Model Organisms Center. All experiments included age- and sex-matched littermate controls, and both males and females aged between 7 and 8 weeks were used. Mice were housed under specific pathogen-free conditions in Central South University and were provided ad libitum access to chow diet (MD17121, Research Diets) and water and housed under a 12-h light/dark cycle. A high-fat diet (HFD) (Teklad TD. 120528) consisting of 17.3% protein, 21.2% fat, 48.5% carbohydrates, and 1.25% cholesterol was used to increase CETP expression and its activity. Protocols were approved by Institutional Ethics Committee for animal procedures of the Central South University (No. CSU-2022-0082).

#### Disuccinimidyl suberate-induced colitis

For the acute colitis model, mice received 1% or 2% indicated disuccinimidyl suberate (DSS; 36,000 to 50,000 molecular weight; MP Biomedicals) in their drinking water for 7 days, followed by 3 days of distilled water without DSS. For the chronic colitis model, mice were supplemented with 2% DSS in their drinking water for 5 days, followed by distilled water without DSS for 10 days, and 3 cycles were repeated. Control animals received distilled water for the entire period. Mice were monitored for body weight and disease activity index score.

#### Evacetrapib treatment

For pharmacological elevation of HDL-C level, 10 mg/kg per day evacetrapib (from Shanghai Bide Pharmatech Ltd., BD301963, 98%) was administered daily in the drinking water for 3 weeks before DSS. The daily dose of the drug was adjusted according to the volume of drunk water and the body weight (measured every week). For rescuing DSS-induced colitis, mice (without evacetrapib in drinking water) were treated with 1 mg/kg evacetrapib intraperitoneal every other day (D0, 2, 4, 6, and 8) and euthanized at D10. For macrophage clearance, clodronate liposome and control liposome (from Liposoma) were intraperitoneally injected once before modeling with 200 μl each mouse.

#### Histology

Distal colon was fixed with 4% paraformaldehyde and embedded in paraffin. Tissue sections (5 μm) were prepared, deparaffinized, and stained with H&E. Histological scores were assigned by experimenters “blinded” to sample identity. Colonic epithelial damage was scored according to previous publication (43). All images were acquired with Leica microscope.

#### Immunohistochemistry and immunofluorescence

Tissue sections were immersed in 3% H_2_O_2_ to block endogenous peroxidase activity, and blocked in 5% BSA for 60 min. Tissues were incubated with anti-TNFα and anti-F4/80 antibodies over night at 4°C, followed by incubation with the conjugated secondary antibodies at room temperature for another 2 h. diaminobenzidine or 4',6-diamidino-2-phenylindole was used to stain the cell nuclei. Alexa Fluor 488 was used to stain F4/80. All images were acquired with Leica microscope.

#### Isolation of intestinal macrophages

Mouse colonic tissues were harvested, cleared of luminal contents, and fragmented. Sequential enzymatic digestion involved an initial 15-min incubation at 37°C in RPMI 1640 medium (Gibco) supplemented with 1 mM DTT (Gibco) and 0.05% FBS (Gibco), followed by a 15-min treatment at 37°C with HBSS (Gibco) containing 0.05% FBS and 5 mM EDTA. Particulate fractions were further digested for 1 h at 37°C with constant agitation in RPMI 1640 medium containing 1 mg/ml collagenase IV (Gibco) and 100 ng/ml DNase I (Gibco). The digested suspension was filtered (70-μm cell strainer), and single cells were isolated by centrifugation of the collected supernatant. Cells were then resuspended in flow cytometry staining buffer (PBS with 2% FBS). Fc receptors were blocked using anti-CD16/CD32 antibodies (BD, 553141) prior to staining with a viability dye and the following antibodies: CD45 APC-Cy7 (BD, 561037), CD11b AF647 (BD, 557686), F4/80 BV421 (BD, 565411), CD86 PE (BD, 567592), and CD206 AF488 (BD, 5688070). Stained samples were analyzed via flow cytometry (BD), and data files were analyzed with FlowJo V10.8.1 software.

#### Quantitative PCR

RNA was isolated with the E.Z.N.A. Total RNA Kit II RNA Isolation Kit (OMEGA), and cDNA was generated with *PerfectStart* Uni real-time quantitative reverse transcription polymerase chain reaction Kit (Transgen), followed by real-time PCR using SYBR Green Master mix with ROX and ABI QuantStudio 7 Flex instrument. real-time quantitative reverse transcription polymerase chain reaction primers sequence was shown in Supplemental Table 2. Gene expression was normalized to *Gapdh*.

#### Western blot

Cells and mice colon tissues were lysed in RIPA lysis buffer to extract protein. The use BCA Protein Analysis Kit (Life-iLab, CHN) to quantify. The proteins were separated by 10% sodium dodecyl sulfate polyacrylamide gel electrophoresis (Life-iLab, CHN) and transferred to a PVDF membrane (Immobilon). The membranes were blocked with 3% BSA (Life-iLab, CHN) for 1 h. Then, the primary antibodies against Atf3 (Thermo Fisher Scientific) and GAPDH (Thermo Fisher Scientific) were used to incubate at 4°C overnight. After washing with Tris-buffered saline containing 0.1% Tween 20 solution, membrane was incubated with HRP-conjugated AffiniPure goat anti-rabbit IgG (Proteintech, CHN) for 1 h.

#### Cell and treatments

Bone marrow–derived macrophage was extracted from WT mouse bone marrow. Cells were stimulated with 50 ng/ml L-4F (HDL mimetics from QYABIO) for 12 h after 4-day culture. Then, cells were restimulated with lipopolysaccharide (20 ng/ml, Solarbio Life Sciences) for 12 h.

### Serum lipids particle number and size analysis

Serum lipids particle number and size of IBD patients were tested by Medical system Laboratory (Ningbo, China). Serum samples were divided into several layers, containing HDL, intermediate density lipoprotein cholesterol (IDL), LDL, VLDL, and Lp(a) by vertical density gradient ultracentrifugation using specially designed centrifuge tubes. The bottom of the centrifuge tube was punctured and inserted into the AU680 autoanalyzer (Beckman Coulter, Brea). The concentration of various lipoprotein cholesterol was quantitatively detected by the continuous density scanning method.

#### Crypt isolation and organoid culture

Colon sections of approximately 5 cm were harvested, opened longitudinally, rinsed with cold PBS, cut into 2–4 mm pieces, and incubated in 2 mM EDTA at 4°C for 40 min with gentle shaking. Crypts were released after vigorous shaking in cold PBS and passed through a 70 μM cell strainer. Approximately 500 crypts were embedded in 20 μl Matrigel (Corning, 356231) and plated in 48-well plates. After Matrigel polymerization, 250 μl IntestiCult Organoid Growth Medium (Stem cell, 06005) was added. For the organoid injury model, we added 5 ng/ml TNFα to the culture medium for 24–48 h.

#### Clinical inflammatory parameters analysis

Patients' blood samples were collected and plasma was isolated according to the manufacturer's protocol. Serum lipid profile, CRP, ESR, fibrinogen, and WBC were analyzed right now at the department of clinical laboratory in our hospital. Aliquoted plasma was stored at −80^o^C until proinflammatory cytokines (TNFα, IL-1β, and IL-6) were analyses by ELISA kit.

#### RNA sequencing library preparation and sequencing

Total RNA was extracted from the tissues using TRIzol® Reagent according the manufacturer's instructions (Invitrogen) and genomic DNA was removed using DNase I (TaKara). RNA purification, reverse transcription, library construction, and sequencing were performed at Shanghai Majorbio Bio-pharm Biotechnology Co., Ltd. (Shanghai, China) according to the manufacturer's instructions (Illumina, San Diego, CA). The transcriptome library was prepared following TruSeq RNA sample preparation Kit from Illumina (San Diego, CA). The raw paired end reads were trimmed and quality controlled by fastp (https://github.com/OpenGene/fastp) with default parameters (44). Then, clean reads were separately aligned to reference genome with orientation mode using HISAT2 (http://ccb.jhu.edu/software/hisat2/index.shtml) software (45). The mapped reads of each sample were assembled by StringTie (https://ccb.jhu.edu/software/stringtie/) in a reference-based approach (46). To identify differentially expressed gene (DEG) between two different groups, the expression level of each gene was calculated according to the transcripts per million reads method. RNA-seg by expectation-maximization (http://deweylab.biostat.wisc.edu/rsem/) was used to quantify gene abundances (47). In addition, functional-enrichment Kyoto Encyclopedia of Genes and Genomes (KEGG) analysis (http://www.genome.jp/kegg/) were performed. KEGG pathway analyses were carried out by Goatools (https://github.com/tanghaibao/Goatools) and KOBAS (http://kobas.cbi.pku.edu.cn/home.do) (48).

#### Statistics

Propensity matching method was used to balance the bias caused by age and sex between control group and IBD patients. Experimental results were analyzed for significance using χ^2^ test, paired or unpaired Student's *t* test, rank-sum test for non-normal quantitative variables as appropriate for comparisons between 2 groups, the Pearson correlation coefficient and ROC curve. Statistical analyses were performed using GraphPad Prism. *P* values are shown as ∗*P* < 0.05, ∗∗*P* < 0.01, ∗∗∗*P* < 0.001, and ∗∗∗∗*P* < 0.0001 where statistical significance was found, and all data are represented as means ± SEM. Individual points in graphs represent biological replicates (i.e., individual mice) pooled from multiple experiments.

## Results

### Serum cholesterol declined in IBD patients

**Fig. 1:**
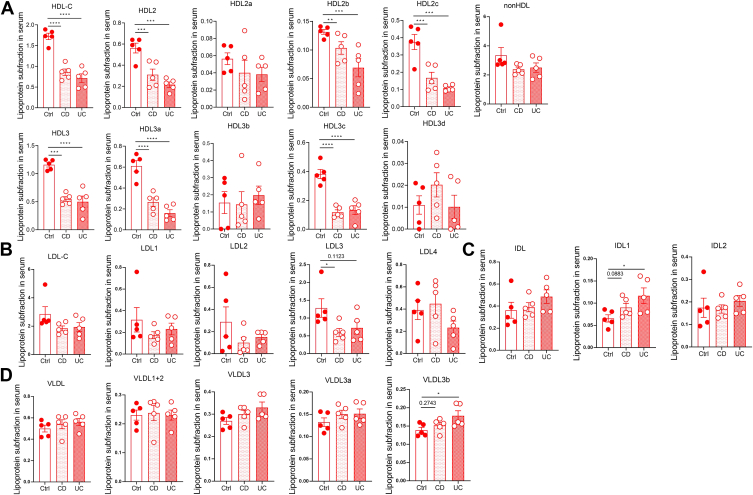
Lipid particle number and size in serum among representative CD and UC patients. A: Level of HDL particle number and size. B: Level of LDL-C particle number and size. C: Level of intermediate density lipoprotein cholesterol (IDL) particle number and size. D: Level of VLDL particle number and size. Data points represent individual patients. *P* values were calculated by paired Student's *t* test; ∗*P* < 0.05, ∗∗*P* < 0.01, and ∗∗∗*P* < 0.001. CD, Crohn's disease; UC, ulcerative colitis.

We first established the relationship between serum lipids and disease activity in our retrospective cohort. Clinical characteristics of 94 patients affected by CD and 79 patients by UC were outlined in Table 1. There was no significant difference in nutritional and inflammatory markers among different Montreal Classification CD patients (Supplemental Table 1). In serum lipids panel, a statistical difference was mainly noted in cholesterol level, with significantly lower TC, HDL-C and LDL-C in both CD and UC patients. No difference was found in the ratio of HDL-C with total cholesterol (TC) or LDL-C. Additionally, we analyzed cholesterol particle number and size in the serum of 5 CD patients, 5 UC patients, and 5 healthy controls. Levels of HDL2, HDL2b, HDL2c, HDL3, HDL3a, and HDL3c were significantly decreased, whereas HDL2a, HDL3b, HDL3d, non-HDL, LDL-C, LDL-1-4, VLDL, VLDL1+2, VLDL3, IDL, IDL1, and IDL2 showed no significant changes in the serum of CD and UC patients (Fig. 1A–D, Supplemental Fig. 4). Publications discussing lipids profile among IBD patients have shown conflicting results, so we performed a meta-analysis to obtain a large sample size. Since CD patients showed significantly lower cholesterol levels than UC patients in our cohort, only publications that reported CD and UC lipid data separately were enrolled for the meta-analysis (423 CD patients and 298 UC patients). Pooled results again demonstrated patients with CD or UC had lower serum cholesterol but unchanged triglycerides (TGs) (Supplemental Figs. 1 and 2). Overall, results from our cohort and meta-analysis all support the conclusion that IBD patients have hypolipidemia, especially lower serum cholesterol level.

**Table 1:** Characteristics of IBD patients and comparable health control

	CD			UC			CD versus UC
	Control (n = 41)	Patients (n = 94)	*P* value	Control (n = 28)	Patients (n = 79)	*P* value	
Age (years)	33.32 (25, 36)	30.44 (14, 66)	0.1237	44.93 (37, 50)	43.72 (14, 72)	0.6812	**<0.0001**
Gender (M/F)	30/11	74/20	0.5090	16/12	46/33	0.9204	**0.0036**
CRP (mg/l)	2.23 (1.07, 6.73)	29.19 (1.00, 282.00)	**<0.0001**	2.02 (1.15, 2.97)	21.86 (1.07, 113.00)	**0.005**	0.1945
Serum lipids							
TG (mmol/l)	1.66 (0.53, 6.61)	1.33 (0.34, 4.10)	0.0522	2.20 (0.64, 7.97)	1.51 (0.51, 10.70)	**0.0325**	0.2043
TC (mmol/l)	4.89 (3.35, 8.28)	3.50 (0.91, 5.49)	**<0.0001**	5.25 (3.87, 7.27)	4.11 (1.64, 7.76)	**<0.0001**	**0.0003**
LDL-C (mmol/l)	3.18 (1.85, 5.36)	2.31 (0.77, 3.89)	**<0.0001**	3.37 (1.86, 5.04)	2.58 (0.92, 4.06)	**<0.0001**	**0.0162**
HDL-C (mmol/l)	1.27 (0.83, 2.05)	0.90 (0.48, 1.49)	**<0.0001**	1.30 (0.68, 2.01)	1.02 (0.43, 2.41)	**0.0005**	**0.0066**
HDL-C/TC	0.27 (0.16, 0.47)	0.28 (0.14, 1.29)	0.6512	0.25 (0.16, 0.51)	0.26 (0.09, 0.48)	0.8302	0.1684
HDL-C/LDL-C	0.43 (0.24, 0.90)	0.42 (0.19, 0.85)	0.6204	0.41 (0.23, 1.08)	0.42 (0.19, 0.85)	0.7232	0.9131

Values are mean (min, max). *P* value by unpaired *t* test for continuous variables and by Fisher's exact test statistic for categorical variables. Bold values are for *P* < 0.05.

IBD, inflammatory bowel disease; CD, Crohn's disease; UC, ulcerative colitis; n, number of patients per group; M/F, male/female; CRP, C-reactive protein; TG, triglyceride; TC, total cholesterol; HDL-C/TC, the ratio of HDL-C with TC; HDL-C/LDL-C, the ratio of HDL-C with LDL-C.

### Serum cholesterol level, especially HDL-C level, was negatively correlated with IBD patients' inflammatory status

Given the significant reduction of cholesterol among IBD patients with active disease, we next sought to elucidate if serum lipids level was associated with level of inflammation. Serum TG level had no correlation with any clinical inflammatory markers, while TC and LDL-C level were inversely correlated with Crohn's disease activity index (CDAI), CRP, and IL-6 in CD patients and with CRP, ESR, IL-1β, and WBC in UC patients (Table 2). Notably, HDL-C levels had statistically significant inverse correlations with CDAI, CRP, fibrinogen, TNFα, IL-6, and WBC in CD patients and with CRP, ESR, and WBC in UC patients. Nevertheless, HDL-C did not show any correlation with BMI, while other serum lipids, like TG, TC, and LDL-C were all significantly correlated with BMI (Table 2). This was further validated in the prospective cohort (Table 3). Patients with normal CRP (<8 mg/ml) had significantly higher HDL-C but no difference of LDL-C, TC, and TG (Supplemental Fig. 3A). Univariate and multivariate regression analyses indicated that HDL-C may serve as a protective factor for IBD (Supplemental Table 2), suggesting that HDL-C could be the most consistent lipid panel marker associated with the inflammatory status of IBD patients.

**Table 2:** Linear correlations between serum lipids and clinical inflammatory markers among IBD patients

	CD											UC								
	CDAI	CRP	ESR	Fibrinogen	TNFα	IL-6	IL-1β	WBC	BMI	ALB	TP	CRP	ESR	Fibrinogen	TNFα	IL-6	IL-1β	WBC	ALB	TP
TG																				
r	−0.0949	−0.0852	−0.1299	−0.1172	−0.083	−0.1116	0.1633	−0.0921	**0.2908**	0.0875	−0.0114	−0.034	−0.203	0.016	−0.258	−0.185	0.465	−0.019	−0.161	−0.236
*P* value	0.3821	0.4145	0.212	0.2605	0.511	0.3761	0.1936	0.3774	**0.0161**	0.4016	0.9135	0.770	0.081	0.895	0.374	0.527	0.150	0.870	0.162	**0.039**
TC																				
r	**−0.375**	**−0.2676**	0.0036	−0.084	−0.2107	**−0.2842**	0.0917	−0.0114	**0.2964**	**0.3459**	**0.2594**	**−0.285**	**−0.361**	−0.056	−0.067	−0.042	**0.787**	**−0.325**	0.049	0.078
*P* value	**0.0004**	**0.0091**	0.9721	0.4208	0.0921	**0.0218**	0.4677	0.2734	**0.0141**	**0.0006**	**0.0116**	**0.013**	**0.001**	0.634	0.821	0.886	**0.004**	**0.004**	0.670	0.499
LDL-C																				
r	**−0.2956**	**−0.257**	−0.0137	−0.1159	−0.2143	**−0.2876**	−0.0569	−0.183	**0.2759**	**0.2802**	**0.2267**	**−0.245**	**−0.239**	−0.047	0.166	−0.081	**0.678**	**−0.359**	0.145	**0.274**
*P* value	**0.0054**	**0.0124**	0.8959	0.2661	0.0865	**0.0202**	0.6527	0.0775	**0.0228**	**0.0062**	**0.028**	**0.033**	**0.039**	0.691	0.570	0.784	**0.022**	**0.001**	0.207	**0.016**
HDL-C																				
r	**−0.2705**	**−0.4586**	−0.1119	**−0.2853**	**−0.2745**	**−0.33**	0.2204	**−0.2186**	−0.0757	**0.4716**	**0.3539**	**−0.378**	**−0.332**	−0.049	−0.029	−0.201	0.407	**−0.244**	0.162	**0.230**
*P* value	**0.0113**	**<0.0001**	0.2829	**0.0053**	**0.0269**	**0.0073**	0.0777	**0.0343**	0.5396	**<0.0001**	**0.0005**	**<0.0001**	**0.004**	0.677	0.921	0.491	0.214	**0.033**	0.159	**0.044**

Pearson r and *P* value. Bold values are for *P* < 0.05.

IBD, inflammatory bowel disease; CD, Crohn's disease; UC, ulcerative colitis; CDAI, Crohn's disease activity index; CRP, C-reactive protein; ESR, erythrocyte sedimentation rate; IL-6: Interleukin 6; IL-1β: Interleukin 1 beta; WBC, white blood count; TG, triglyceride; TC, total cholesterol; BMI, body mass index; ALB, albumin; TP, serum total protein.

**Table 3:** HDL-C level at admission predicts CD patients’ outcomes

Admission	n	1 month		n	2 months		n	4 monthss	
		CDAI	CRP (mg/l)		CDAI	CRP (mg/l)		CDAI	CRP (mg/l)
HDL-C (mmol/l)									
<0.9050	16	164.9 (30.95, 704.08)	6.586 (1.20, 21.70)	16	136.6 (40.03, 329.28)	6.398 (2.05, 13.20)	12	129.8 (27.16, 378.00)	9.459 (1.24, 25.20)
≧0.9050	19	102.4 (6.90, 193.93)	7.377 (1.01, 76.80)	15	83.55 (20.35, 234.88)	1.547 (0.21, 2.80)	14	73.56 (38.46, 145.57)	3.151 (1.00, 11.10)
*P* value		0.1305	0.8689		**0.0417**	**0.0007**		0.0597	**0.0157**
CRP (mg/l)									
<8	10	107.5 (52.32, 193.93)	4.894 (1.14, 24.80)	9	101.1 (20.35, 277.19)	3.021 (1.05, 3.02)	8	85.49 (38.46, 145.57)	2.526 (1.16, 4.58)
≧8	25	138.1 (6.90, 704.08)	7.884 (1.01, 76.80)	22	112.8 (50.77, 329.28)	4.472 (0.21, 13.2)	18	102.7 (27.16, 378.00)	7.634 (1.00, 22.8)
*P* value		0.5166	0.5681		0.6996	0.4022		0.5948	0.0782

Values are mean (min, max). *P* value by unpaired *t* test for continuous variables.

Bold values are for *P* < 0.05.

CD, Crohn’s disease; n, number of patients per group; CRP, C-reactive protein; CDAI, Crohn's disease activity index.

Next, we questioned if HDL-C level at admission could predict patient outcome. Based on CRP level (clinical cut-off: 8 mg/ml), we found 0.9050 mmol/l of HDL-C was a reasonable cut-off with relatively good sensitivity, specificity, and likelihood ratio (Supplemental Fig. 3B and C). Interestingly, CD patients with relatively lower HDL-C at admission were more likely to have higher CDAI and CRP at the 2- and 4-month follow-up (2-month CRP: mean ± SD, 6.398 ± 4.925 versus 1.547 ± 0.713 mg/l, unpaired *t* test, *P =* 0.0007; 4-month CRP: mean ± SD, 9.459 ± 8.530 versus 3.151 ± 2.932 mg/l, unpaired *t* test, *P =* 0.0157. Table 4). In contrast, CRP, which is currently considered the best clinical inflammatory marker for IBD patients, did not show good predictive value for future disease activity. Collectively, these findings indicate that HDL-C may serve as a marker to reflect and predict IBD patient inflammatory status, perhaps indicating which patients would most benefit from more aggressive therapeutic intervention.

**Table 4:** HDL-C level at admission predicts CD patients' outcomes

Admission	n	1 month		n	2 months		n	4 months	
		CDAI	CRP (mg/l)		CDAI	CRP (mg/l)		CDAI	CRP (mg/l)
HDL-C (mmol/l)									
<0.9050	16	164.9 (30.95, 704.08)	6.586 (1.20, 21.70)	16	136.6 (40.03, 329.28)	6.398 (2.05, 13.20)	12	129.8 (27.16, 378.00)	9.459 (1.24, 25.20)
≧0.9050	19	102.4 (6.90, 193.93)	7.377 (1.01, 76.80)	15	83.55 (20.35, 234.88)	1.547 (0.21, 2.80)	14	73.56 (38.46, 145.57)	3.151 (1.00, 11.10)
*P* value		0.1305	0.8689		**0.0417**	**0.0007**		0.0597	**0.0157**
CRP (mg/l)									
<8	10	107.5 (52.32, 193.93)	4.894 (1.14, 24.80)	9	101.1 (20.35, 277.19)	3.021 (1.05, 3.02)	8	85.49 (38.46, 145.57)	2.526 (1.16, 4.58)
≧8	25	138.1 (6.90, 704.08)	7.884 (1.01, 76.80)	22	112.8 (50.77, 329.28)	4.472 (0.21, 13.2)	18	102.7 (27.16, 378.00)	7.634 (1.00, 22.8)
*P* value		0.5166	0.5681		0.6996	0.4022		0.5948	0.0782

Values are mean (min, max). *P* value by unpaired *t* test for continuous variables.

Bold values are for *P* < 0.05.

CD, Crohn's disease; n, number of patients per group; CDAI, Crohn's disease activity index; CRP, C-reactive protein.

### Anti-TNFα therapy elevated HDL-C level in responding CD patients

**Fig. 2:**
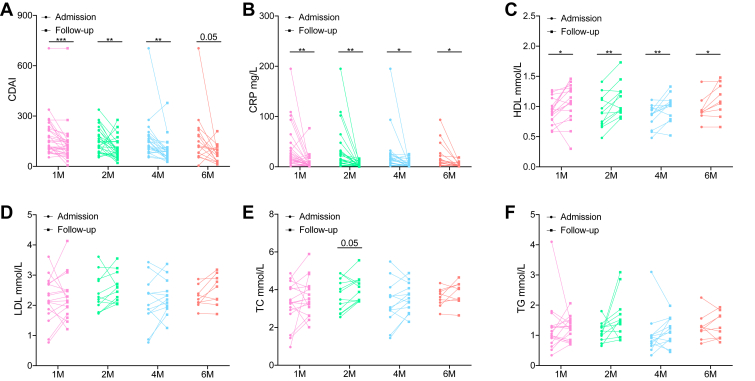
Clinical inflammatory marker and serum lipid profile among CD patients after anti-TNFα treatment. A: Crohn's disease activity index (CDAI). B: C-reactive protein (CRP). C: Serum HDL-C level. D: Serum LDL-C level. E: Serum total cholesterol (TC) level. F: Serum triglyceride (TG) level. Data points represent individual patients. *P* values were calculated by paired Student's *t* test; ∗*P* < 0.05, ∗∗*P* < 0.01, and ∗∗∗*P* < 0.001. CD, Crohn's disease.

To validate whether HDL-C level could be a useful parameter for evaluating inflammatory status in IBD patients, we followed anti-TNFα (infliximab) treated CD patients up to 6 months. Infliximab evidently improved CD patients' symptoms and 1-month treatment alleviated colitis with significant reduction in CDAI and CRP (Fig. 2A and B). Likewise, substantially increased HDL-C level compared to baseline was found during 1-, 2-, 4- and 6-month follow-up (Fig. 2C). Notably, one infliximab nonresponder who had increased CDAI and CRP at 1-month follow-up shown decreased HDL-C at the same time, which further supported the idea that HDL-C level closely related to inflammation. Unlike HDL-C, none of LDL-C, TC nor TG were elevated despite colitis improvement (Fig. 2D–F). All in all, improved colitis following anti-TNFα therapy in CD patients was associated with increased HDL-C levels.

### CETPi prevented experimental colitis progression

**Fig. 3:**
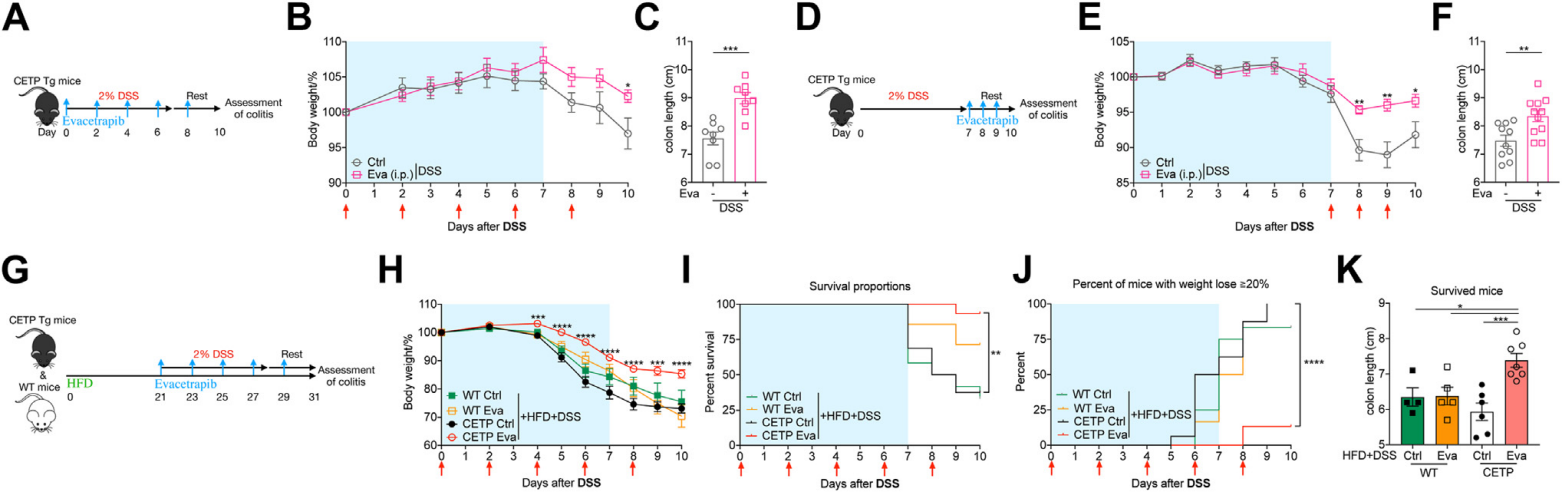
CETPi prevented experimental colitis progression. A–C: CETP-Tg male mice were fed on chow diet and got evacetrapib by intraperitoneal injection every other day and outcomes were analyzed after DSS treatment. A: Experimental design. B: Body weights shown as percentage of starting weight (n = 8 per group). C: Colon length. D–F: CETP-Tg male mice were fed on chow diet and got evacetrapib by intraperitoneal injection every day since D7 after DSS and outcomes were analyzed on D10. D: Experimental design. E: Body weights shown as percentage of starting weight (n = 10 to 11 per group). F: Colon length. G–K: CETP–Tg and WT male mice were fed on HFD for 3 weeks and got evacetrapib by intraperitoneal injection every other day once started DSS. Outcomes were analyzed after DSS treatment. G: Experimental design. H: Body weights shown as percentage of starting weight (n = 7 to 16 per group). I: Survival curve. J: Proportion of mice with more than 20% weight lost. K: Colon length of survived mice. Data points represent individual mice, pooled from two independent experiments. All data are represented as means ± SEM. *P* values were calculated by Student's *t* test or one-way ANOVA. For (B), (E), and (H), Student's *t* test was performed independently at each time point; ∗*P* < 0.05, ∗∗*P* < 0.01, ∗∗∗*P* < 0.001, and ∗∗∗∗*P* < 0.0001. CETPi, cholesteryl ester transfer protein inhibitor; CETP-Tg, CETP transgenic; DSS, disuccinimidyl suberate; HFD, high-fat diet.

The results so far show clear associations between HDL-C and inflammatory status in IBD, leading us to ask if increasing HDL-C could be a potential pharmacological target for IBD patient therapy. Therefore, we tested the hypothesis that administration of evacetrapib, a third generation CETPi, at the onset of or even after experimental colitis would attenuate disease progression. First, CETP transgenic (CETP-Tg) mice received evacetrapib intraperitoneal injection every other day after DSS administration (Fig. 3A). As expected, CETP-Tg mice treated with evacetrapib showed a better colitis outcome, with less weight loss and longer colon length (Fig. 3B and C). We next asked whether evacetrapib could contribute in the recovery phase of colitis (Fig. 3D). In this context, evacetrapib significantly accelerated the recovery of mice against colitis relative to that in the control group (Fig. 3E and F).

We repeated this study in mice fed on a 3-week HFD prior to DSS (Fig. 3G), which results in a more robust HDL-C response to CETPi (49). HFD increases sensitivity to DSS colitis due to changes in microbiota composition (50), hence it was expected that a more striking weight loss occurred in these experiments (Fig. 3H) leading to a survival rate of only ∼50% of mice by the end of experiment (Fig. 3I). Nevertheless, CETP-Tg mouse survival was significantly improved by treating with evacetrapib (Fig. 3I and J). Besides, colon length among surviving mice was much longer in evacetrapib-treated CETP-Tg mice (Fig. 3K). All in all, acute pharmacological CETPi intervention after the onset of colitis allayed disease severity.

### Evacetrapib improved colitis in WT mice

**Fig. 4:**
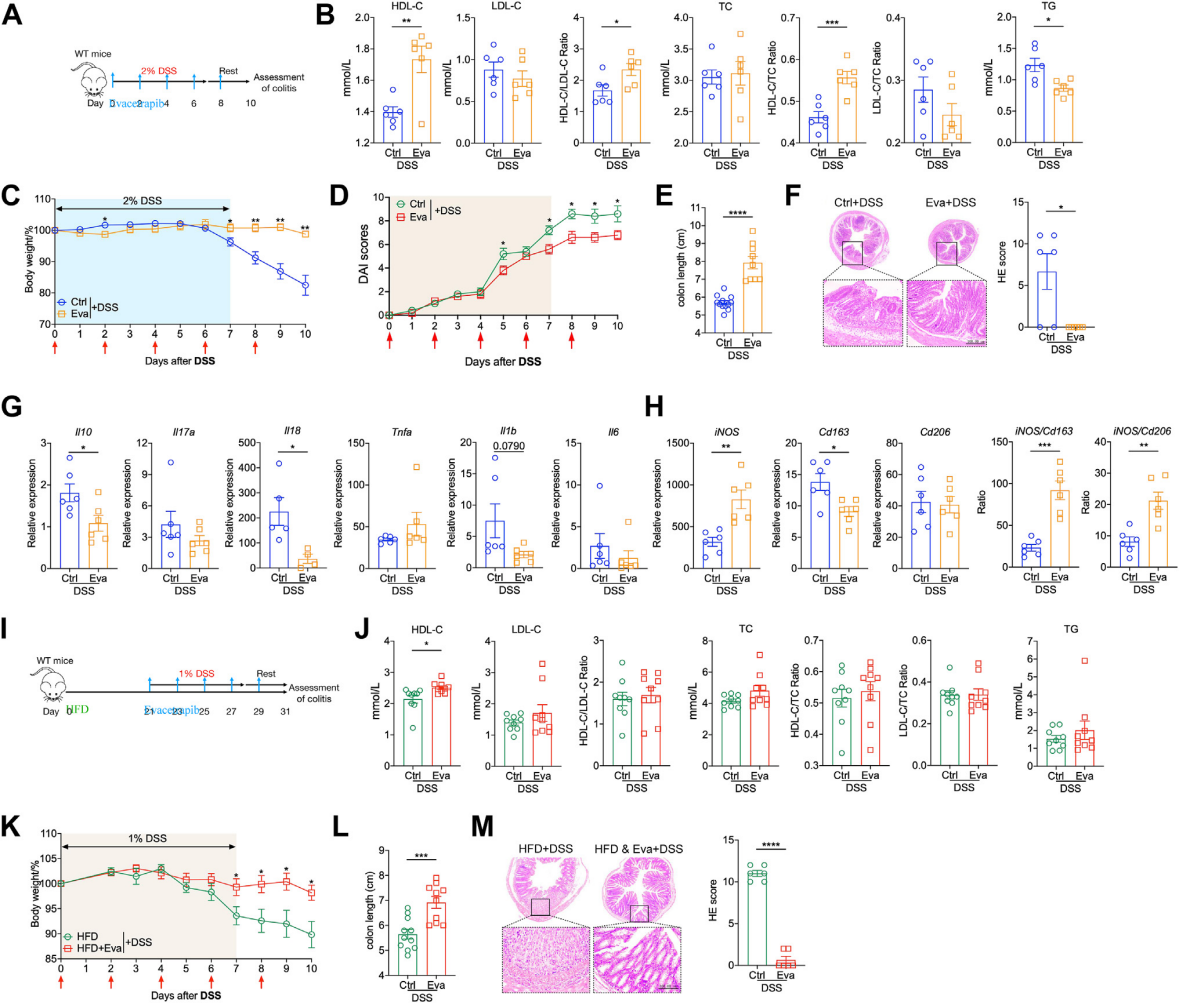
Evacetrapib improved colitis in WT mice. A–G: WT male mice were fed on chow diet and got evacetrapib by intraperitoneal injection every other day and outcomes were analyzed after DSS treatment. A: Experimental design. B: Serum HDL-C, LDL-C, total cholesterol, triglyceride level, and their ratio. C: Body weights shown as percentage of starting weight (n = 9 to 12 per group). D: Disease activity index score. E: Colon length. F: Representative images of H&E of tissue sections from distal colon and histological score. G and H: Expression of indicated genes in distal colon tissue, normalized to *Gapdh*. I to M: WT male mice were fed on HFD and got evacetrapib by intraperitoneal injection every other day and outcomes were analyzed after DSS treatment. I: Experimental design. J: Serum HDL-C, LDL-C, total cholesterol, triglyceride level and their ratio. K: Body weights shown as percentage of starting weight (n = 10 to 11 per group). L: Colon length. M: Representative images of H&E of tissue sections from distal colon and histological score. Data points represent individual mice, pooled from two independent experiments. All data are represented as means ± SEM. *P* values were calculated by Student's *t* test. For (C) and (J), Student's *t* test was performed independently at each time point; ∗*P* < 0.05, ∗∗*P* < 0.01, ∗∗∗*P* < 0.001, and ∗∗∗∗*P* < 0.0001. DSS, disuccinimidyl suberate.

**Fig. 5:**
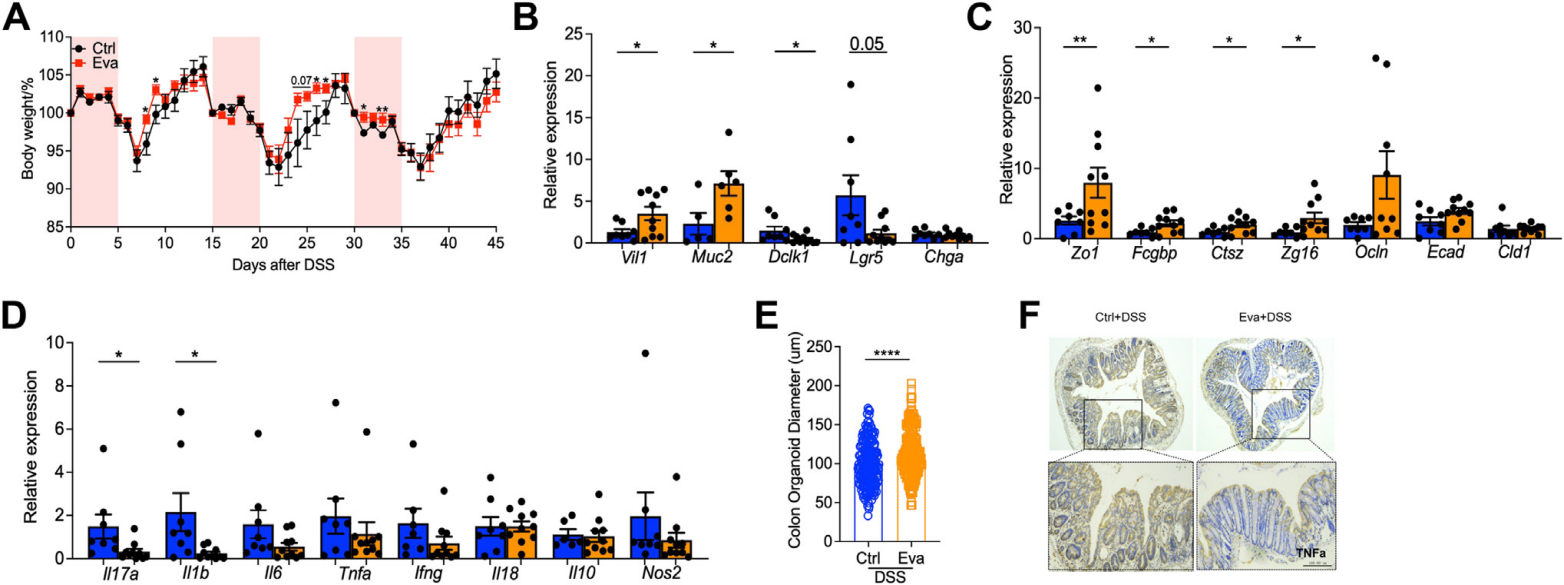
Evacetrapib improved chronic colitis in WT mice. A–F: WT male mice were fed on chow diet and got evacetrapib by intraperitoneal injection every other day and outcomes were analyzed after three cycles of DSS treatment. A: Body weights shown as percentage of starting weight (n = 5 per group). B–D: Expression of indicated genes in distal colon tissue, normalized to Gapdh. E: Colon organoid size. Scale bar 100 μm. F: Representative images of immunohistochemistry of TNFα expression in tissue sections from distal colon. DSS, disuccinimidyl suberate.

WT mice naturally have low CETP expression and activity, thus they are considered nonresponsive to CETPi and most studies use only CETP-Tg mice (51). However, when percent of survival was analyzed, evacetrapib-treated WT mice shown an unexpected better outcome than their control (Fig. 3H). To further investigate the effect of evacetrapib on WT mice HDL-C and colitis outcome, the same experiment setting was adopted in WT mice (Fig. 4A) and serum lipids profile was analyzed. Mice treated with evacetrapib intraperitoneal injection had selectively elevated HDL-C level (mean: 1.73 versus 1.40; unpaired *t* test, *P = 0*.*0042*. Figure 4B) and its ratio with other lipids, but no clear influence on LDL-C, TC, and TG level. WT mice treated with evacetrapib showed better colitis outcome with less weight loss (Fig. 4C), lower DAI score (Fig. 4D), longer colon length (Fig. 4E), and less damage in colon (Fig. 4F) when challenged with DSS. The effects of evacetrapib on WT mice HDL-C elevation and colitis protection were unexpected but interesting. Then, the effect of evacetrapib on immune-related cytokines (e.g. *Il10*, *Il17a*, *Tnfa*, *Il1b*, *Il6*, *Il18*, and *Ifng*), immune cell infiltration (e.g. *Cd3a*, *iNOS*, *Cd163*, and *Cd206*), enterocytes (e.g. *Dclk1*, *Vil1*, *Chga*, *Muc2* and *Lgr5*), and gut barrier function (e.g. *Fcgbp*, *Zg16*, *Clac1*, *Ctsz*, *Ocln*, *Zo1*, *Cldn1*, and *Ecad*) were explored in colon. Mice receiving evacetrapib injection after DSS administration had lower *Il10* and *Il18* expression (Fig. 4G). In addition, we found a clear effect of evacetrapib on macrophage polarization as shown by higher *iNOS* expression, *iNOS/Cd163*, and *iNOS/Cd206* ratio, without influence on other types of cell (Fig. 4H). The protective effect of evacetrapib was also observed in the chronic colitis model (Fig. 5), manifested in higher body weight, enhanced stem cell activity (e.g. *Vil1*, *Muc2*, *Dclk1*, *Lgr5*, and organoid diameter), better gut barrier function (e.g. *Zo1*, *Fcgbp*, *Ctsz*, and *Zg16*), and lower proinflammatory factor (e.g. *Il17a*,*Il1b* and *TNFa*).

The unexpected protective effect of evacetrapib on WT mice was further tested under HFD conditions. As HFD-fed mice are more sensitive to DSS-induced colitis and experienced low survival with the standard 2% DSS treatment (Fig 3I), 1% DSS was adopted which induced clear colitis in HFD mice (Fig. 4I). Like mice fed on chow diet, we observed a directionally same effects of evacetrapib on serum lipids with only HDL-C influenced (Fig. 4J). Besides, mice fed on HFD also phenocopied the improvement in colitis outcome if mice received evacetrapib treatment (Fig. 4K–M). That is, evacetrapib consistently improved colitis outcome in both CETP-Tg mice and WT mice regardless of diet via selectively increasing HDL-C.

### Pharmacological elevation of HDL-C by evacetrapib pretreatment prevented colitis

**Fig. 6:**
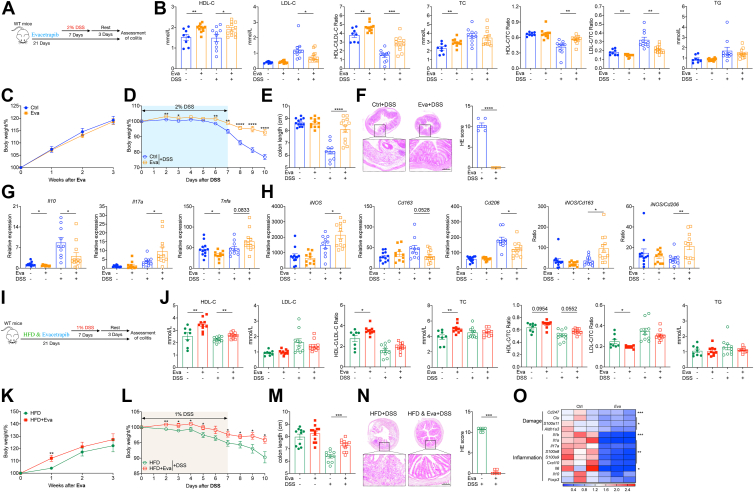
Pharmacological elevation of HDL-C by evacetrapib pretreatment prevented experimental colitis. A–H: WT male mice were fed on chow diet supplied with evacetrapib in drinking water and outcomes were analyzed before or after DSS treatment. A: Experimental design. B: Serum HDL-C, LDL-C, total cholesterol, triglyceride level and their ratio. C: Body weights shown as percentage of starting weight (n = 18 to 24 per group). D: Body weights shown as percentage of starting weight (n = 10 to 12 per group). E: Colon length. F: Representative images of H&E of tissue sections from distal colon and histological score. G and H: Expression of indicated genes in distal colon tissue, normalized to *Gapdh*. I–O: WT male mice were fed on HFD supplied with evacetrapib in drinking water and outcomes were analyzed before or after DSS treatment. I: Experimental design. J: Serum HDL-C, LDL-C, total cholesterol, triglyceride level and their ratio. K: Body weights shown as percentage of starting weight (n = 19 to 23 per group). L: Body weights shown as percentage of starting weight (n = 10 to 11 per group). M: Colon length. N: Representative images of H&E of tissue sections from distal colon and histological score. O: Heat map of damage and inflammation markers expression. Data points represent individual mice, pooled from two independent experiments except for (C) and (K) from four experiment. All data are represented as means ± SEM. *P* values were calculated by Student's *t* test. For (C), (D), (K), and (L), Student's *t* test was performed independently at each time point; ∗*P* < 0.05, ∗∗*P* < 0.01, ∗∗∗*P* < 0.001, and ∗∗∗∗*P* < 0.0001. DSS, disuccinimidyl suberate; HFD, high-fat diet.

To further investigate the colitis prevention potential, evacetrapib was supplied to WT mice (Fig. 6A). After 3 weeks of evacetrapib treatment and before DSS exposure, mice treated with evacetrapib had slightly but significantly higher levels of HDL-C relative to mice treated with placebo (mean: 1.97 versus 1.53 mmol/l; unpaired *t* test, *P =* 0.0064. Fig. 6B). However, there was no significant difference in LDL-C levels between CETPi- and placebo-treated mice (mean: 0.40 versus 0.42 mmol/l; unpaired *t* test, *P =* 0.3819). After DSS administration, we confirmed higher HDL-C level but also lower LDL-C level in evacetrapib treated mice. Notably, evacetrapib constantly increased the ratio of HDL-C/LDL-C regardless of DSS administration (Fig. 6B). Evacetrapib pretreatment only had no obvious effects on body weight (Fig. 6C) and colon length (Fig. 6E), but mice supplied with evacetrapib displayed considerably less severe symptoms than placebo mice in DSS colitis model, reflected by the body weight loss (Fig. 6D), colon length (Fig. 6E), and colon pathology (Fig. 6F). Evacetrapib treatment induced significantly less *Il10* and *Tnfa* expression even at baseline before DSS (Fig. 6G, Supplemental Fig. 5). In addition, changes on macrophages markers by evacetrapib were further confirmed in this preventive model (Fig. 6H).

We also repeated this study in mice fed on HFD (Fig. 6I). HFD feeding increased serum cholesterol level significantly (LDL-C: 133% increase, HDL-C: 66% increase, TC: 71% increase) but had no obvious effects on TG (Fig. 6J). Under HFD feeding, HDL-C level elevated by evacetrapib was much clearer than under chow diet feeding (40% versus 28%), but still evacetrapib had no influence on LDL-C level, suggesting a selective effect of third generation of CETPi (Fig. 6J). CETPi-treated mice gained more weight in the first week, but reached comparable weight (Fig. 6K) and colon length (Fig. 6M) by the end of 3-week administration. As before, we observed improvement in colitis outcome among evacetrapib-pretreated mice under HFD (Fig. 6L–N). RNA sequencing and differential gene expression analysis of colon tissue indicated less damage and inflammation in evacetrapib-treated mice (Fig. 6O). In sum, elevated HDL-C level achieved by evacetrapib administration induced an anti-inflammatory condition that protected mice from experimentally induced colitis.

### Evacetrapib promoted anti-inflammatory reprogramming of macrophages by inducing transcriptional repressor ATF3 and enhanced gut barrier function

**Fig. 7:**
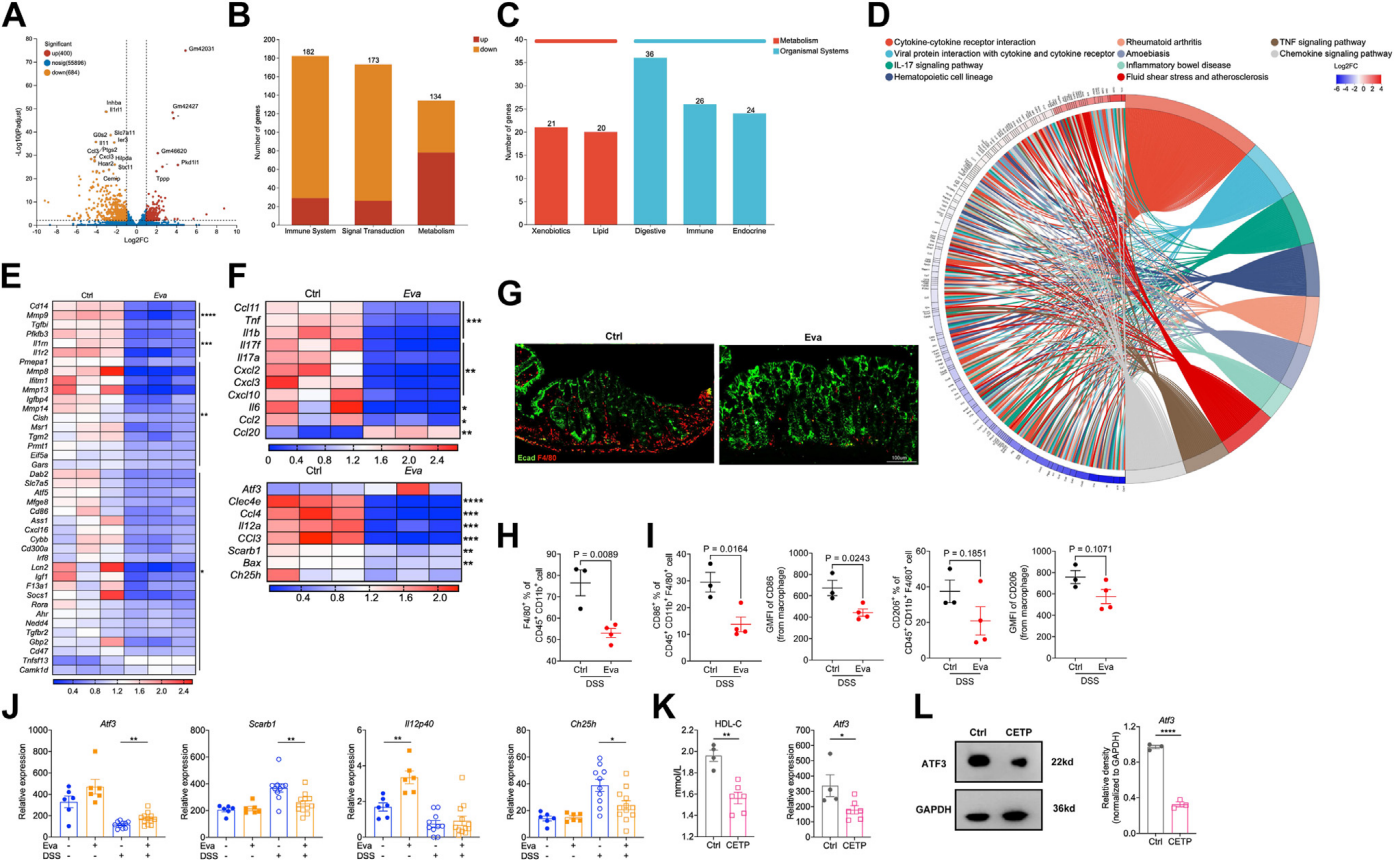
Evacetrapib promoted anti-inflammatory reprogramming of macrophages by including transcriptional repressor ATF3. A–H: WT male mice were fed on HFD supplied with evacetrapib in drinking water and colon tissue RNA sequencing was done after DSS treatment. A: Volcano plot of DEG. B: reactome annotation of DEG. C: KEGG annotation of DEG. D: KEGG enrichment of DEG. E: Heat map of common genes involved in top pathway. F: Heat map of M1 and M2 macrophages markers expression, *Atf3* and its target genes. G: Representative images of F4/80+ macrophage of tissue sections from distal colon. H: Percentage of F4/80^+^ cells in CD45^+^CD11b^+^ cells. I: Percentage of CD86^+^; CD206^+^ cells in F4/80^+^CD11b^+^ cells. J–L: CETP transgenetic mice were fed on chow diet and baseline characteristics were analyzed before DSS treatment. J: Expression of *Atf3* and its target genes. K: Serum HDL-C level and expression of *Atf3* in distal colon tissue, normalized to *Gapdh*. L: Western blotting for ATF3 in distal colon tissue, normalized to *Gapdh*. Data points represent individual mice, pooled from two independent experiments. All data are represented as means ± SEM. *P* values were calculated by Student's *t* test; ∗*P* < 0.05, ∗∗*P* < 0.01, ∗∗∗*P* < 0.001, and ∗∗∗∗*P* < 0.0001. CETP, cholesteryl ester transfer protein; DEG, differentially expressed gene; DSS, disuccinimidyl suberate; HFD, high-fat diet; KEGG, Kyoto Encyclopedia of Genes and Genomes.

To understand the mechanisms of evacetrapib action on colitis, RNA sequencing was performed. We harvested colon tissues from HFD and evacetrapib-pretreated mice after DSS treatment (Fig. 6I). Compared to Ctrl mice, evacetrapib treatment upregulated 400 genes while downregulated 684 genes (Fig. 7A, *P*-adjust < 0.01, DEG fold change >2). Reactome annotation indicated the top three influenced biological process are immune system, signal transduction, and metabolism (Fig. 7B). KEGG annotation showed the main changes in metabolism were related to lipid metabolism, confirming the activity of CETPi inhibitor, and the top-influenced organismal systems were digestive and immune system (Fig. 7C). KEGG enrichment analysis of DEG identified genes involved in cytokine-cytokine receptor interaction, IL-17 signaling pathway, hematopoietic cell lineage, IBD, and TNF signaling pathway were enriched (Fig. 7D). The common genes involved in those pathways were further analyzed and a clue of macrophage contribution was found, like *Tnf*, *Il1b*, *Cxcl2*, *and Cxcl10* (Fig. 7E). Next, markers of M1 and M2 macrophages were analyzed and significant changes were confirmed in RNA sequencing data (Fig. 7F), consistent with our previous real-time PCR findings (Fig 4G and Fig 6G). Based on those results, macrophage staining by F4/80 in colon was adopted, which further confirmed the transcriptional finding by showing less macrophage infiltration in evacetrapib-treated mice (Fig. 7G). Flow cytometry analysis was conducted to evaluate immune cell populations. No significant differences were observed in the proportions of CD45^+^ or CD11b^+^ immune cells. However, treatment with evacetrapib significantly reduced the frequency of CD45^+^CD11b^+^F4/80^+^ macrophages. Notably, the proportion of proinflammatory M1 macrophages (CD86^+^CD11b^+^F4/80^+^ cells) was significantly decreased, whereas the M2 macrophage phenotype (CD206^+^CD11b^+^F4/80^+^ cells) remained unaffected (Fig 7H and I). Nardo *et al*. reported that the broad anti-inflammatory and metabolic actions of HDL-C relied on the transcriptional repressor ATF3 in macrophages under TLR agonists challenged (36). Upregulated expression of *Atf3* and corresponding downregulation of ATF3 target genes were observed in both HFD and chow diet feeding mice in evacetrapib treatment group (Fig. 7J), supporting a role of HDL-ATF3 axis in our mice model. On the other hand, decreasing serum HDL-C level conferred in CETP-Tg mice (Fig. 7K) inhibited *Atf3* expression (Fig. 7L), further confirming the relationship of HDL-C to ATF3.

**Fig. 8:**
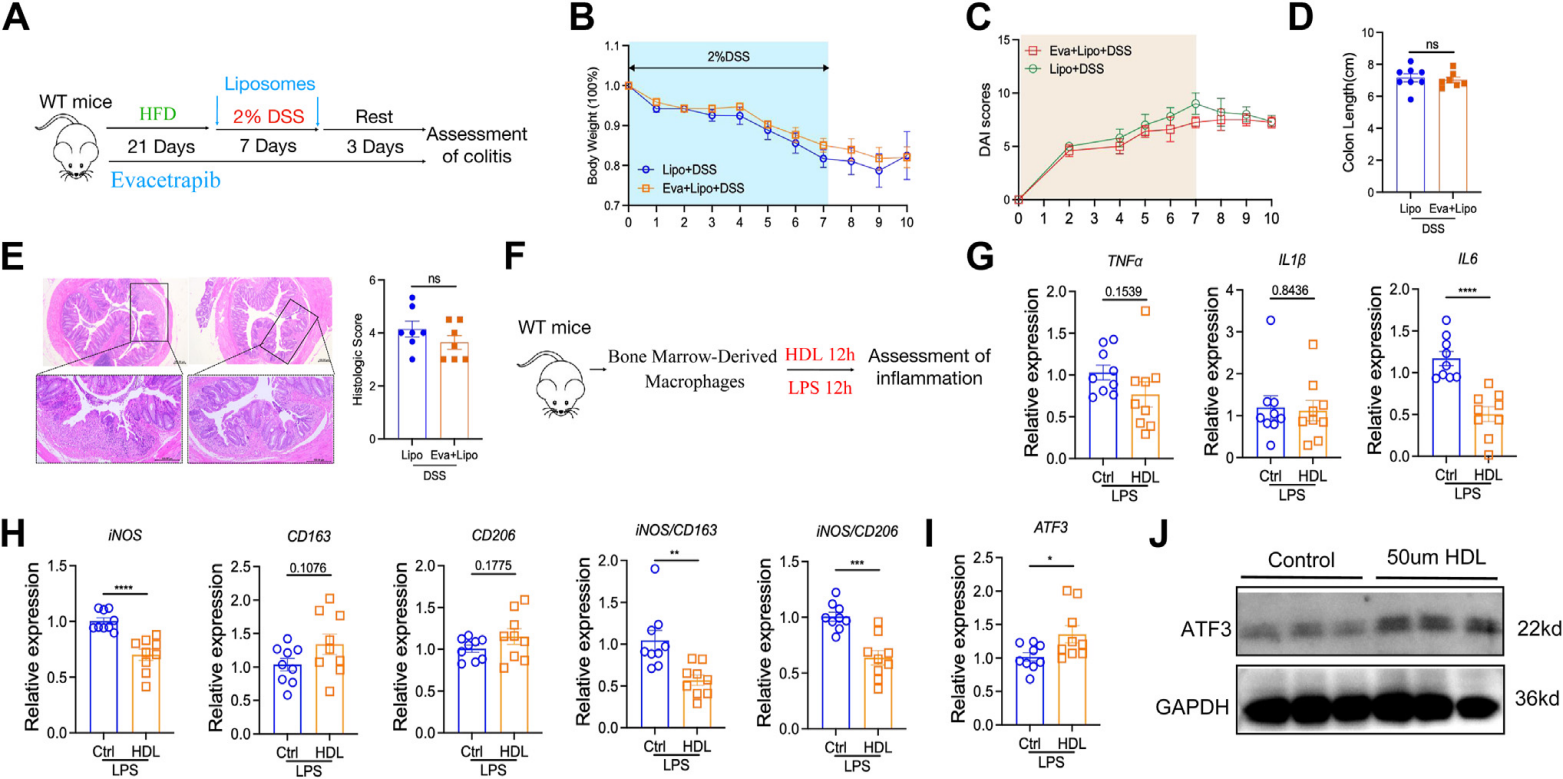
HDL promoted anti-inflammatory reprogramming of macrophages by including transcriptional repressor ATF3. A–E: WT male mice were fed on HFD supplied with evacetrapib in drinking water and outcomes were analyzed after DSS treatment. A: Experimental design. B: Body weights shown as percentage of starting weight (n = 5 per group). C: Disease activity index score. D: Colon length. E: Representative images of H&E of tissue sections from distal colon and histological score. F–J: Bone marrow–derived macrophages was extracted from WT mice and got HDL treatment and outcomes were analyzed after lipopolysaccharide stimulation. G and H: Expression of indicated genes in macrophage, normalized to Gapdh. I and J: Expression of ATF3 in macrophage, normalized to Gapdh. Data points represent individual mice, pooled from two independent experiments. All data are represented as means ± SEM. *P* values were calculated by Student's *t* test; ∗*P* < 0.05, ∗∗*P* < 0.01, ∗∗∗*P* < 0.001, and ∗∗∗∗*P* < 0.0001. DSS, disuccinimidyl suberate; HFD, high-fat diet.

To investigate the role of HDL and macrophages in evacetrapib-induced colitis remission, we administered clodronate-liposome treatment to deplete macrophages (Fig. 8A). Following this treatment, evacetrapib's ability to alleviate colitis was abolished, as evidenced by no significant differences in body weight (Fig. 8B), disease activity index score (Fig. 8C), colon length (Fig. 8D), or H&E staining (Fig. 8E). Furthermore, treatment of bone marrow–derived macrophages with HDL mimetics significantly reduced proinflammatory macrophage activity, demonstrated by decreased IL-6 and iNOS expression, as well as lower iNOS/Cd163 and iNOS/Cd206 ratios (Fig. 8G and H). Additionally, elevating HDL levels markedly upregulated Atf3 expression (Fig. 8I and J).

**Fig. 9:**
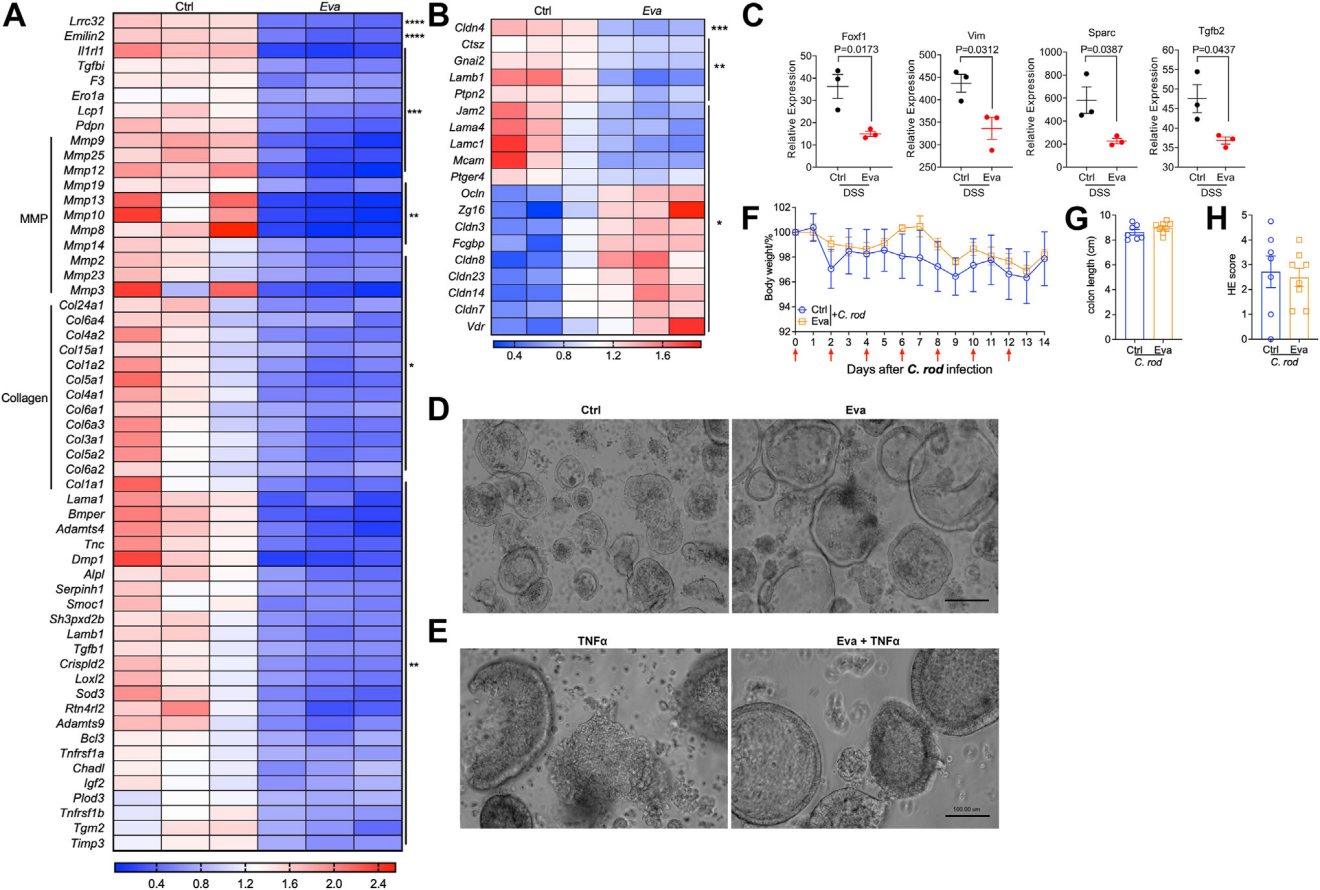
Evacetrapib enhanced gut barrier function. A–C: WT male mice were fed on HFD supplied with evacetrapib in drinking water and outcomes were analyzed after DSS treatment. A: Heat map of genes involved in extracellular matrix remodeling. B: Heat map of genes involved in gut barrier function. C: Expression of genes involved in epithelial-mesenchymal transition. D: Colon organoid size. Scale bar 100 μm. E: Colon organoid appearance after TNFα treatment. F–H: WT mice were fed on chow diet and got evacetrapib by intraperitoneal injection every other day and outcomes were analyzed after *C*. *rodentium* infection. F: Weight length. G: Colon length. H: H&E score. Data points represent individual mice, pooled from two independent experiments. All data are represented as means ± SEM. *P* values were calculated by Student's *t* test; ∗*P* < 0.05, ∗∗*P* < 0.01, ∗∗∗*P* < 0.001, and ∗∗∗∗*P* < 0.0001. DSS, disuccinimidyl suberate; HFD, high-fat diet.

Except for macrophage reprogramming, significant changes in extracellular matrix remodeling (Fig. 9A), gut barrier function (Fig. 9B), and epithelial-mesenchymal transition (Fig. 9C) were found by RNA sequencing, suggesting gut barrier may also be involved for evacetrapib protection on DSS-induced colitis. To further test, in vitro gut organoid was adopted. Evacetrapib-treated colon organoids were significantly larger (Fig. 9D), and the resistance to injury was significantly enhanced (Fig. 9E), demonstrating improved gut barrier function also contributed to better outcome for injury induced colitis. This is extremely critical as we found evacetrapib could not protect mice from infection-induced colitis (Fig. 9F–H), although genes related to gut barrier were upregulated (Supplemental Fig. 6). Together, these data suggest that evacetrapib protects mice from colitis via upregulating ATF3-promoted anti-inflammatory transcriptional reprogramming of macrophages as well as enhanced gut barrier function.

## Discussion

In the present study, results from our cohort and meta-analysis supported the conclusion that lower HDL-C levels in IBD patients are negatively associated with inflammatory status. Accordingly, restored HDL-C level was observed after biologic therapy. This cohort included patients with severe untreated IBD requiring hospital admission, and notably HDL-C levels had better predictive values for patient outcome than the most commonly used clinical inflammatory biomarker CRP. Furthermore, pharmacological elevation of HDL-C level allayed disease severity in an experimental colitis model where mice consumed normal chow diet or HFD. Mechanistically, CETPi increased expression of ATF3, a transcriptional repressor for proinflammatory cytokines, and induced macrophage reprogramming and enhanced gut barrier function. Together, the important advance of our study is the demonstration that HDL-C is a potential modulator of inflammation in IBD patients and pinpoint the pharmacological effect of CETPi, such as evacetrapib, for future IBD therapy.

Acute or chronic systemic inflammation is known to trigger changes in serum lipid and lipoproteins (52). Numerous studies have shown low HDL-C but high TG in inflammatory and autoimmune diseases, including sepsis, IBD, systemic lupus erythematosus, and rheumatoid arthritis (12, 53, 54), and cytokines like TNFα, IL-1β, and IL-6 are thought to be involved (55). Mechanistically, those proinflammatory cytokines could be detected by hepatocytes, resulting in reduced expression and secretion of ApoAI (56). Besides, inflammation may affect the gut microbiota, which not only is associated with blood HDL-C levels but also plays important roles in HDL-C function (57). In this study, we confirmed decreased HDL-C concentration in active IBD patients, especially among CD patients. At the same time, HDL-C level was restored after anti-TNFα therapy, further demonstrating the association of inflammation and HDL-C. But different from previous reports, TG showed no difference among CD patients while significantly lower among UC patients in our cohort. The published relationship between inflammation and LDL-C level is even less clear. IBD patients had significantly lower LDL-C level in our cohort, but in mice DSS treatment increased LDL-C concentration dramatically. As DSS treatment is an acute colitis model, higher LDL-C level may suggest a pathological role of LDL-C in the early stage of colitis. Although not in IBD, there were studies reported an increased LDL-C level in patients with gastrointestinal inflammatory disease, such as coeliac disease (58), *Helicobacter pylori* infection (59) and *Vibrio cholerae* infection (60), despite concurrently reduced HDL-C. Therefore, the contribution and relation of LDL-C in inflammatory diseases could be meaningful to analyze at the onset stage of disease but perhaps less predictive during chronic phases.

While previous studies have investigated lipid profiles in IBD, no reports have explored the relationships between blood lipids and serum inflammatory cytokines, different lipid particle, or the specific role of serum lipids in predicting clinical outcomes. Actually, published findings report the predictive role of HDL-C in sepsis survival (34), acute pancreatitis organ failure (61), and clinically manifest CVD (62). Interestingly, higher HDL-C levels during treatment also predict improved response and survival in CRC patents receiving adjuvant chemotherapy (63). It is notable that our results place clinical meaning to the potential of testing HDL-C at admission to better predict IBD disease outcome, perhaps more accurately than CRP. In addition, HDL-C was the only lipid panel readout restored by anti-TNFα therapy, even at 1-month in responding patients. As HDL-C could act as a marker to reflect and even predict patient inflammatory status, and the expense for testing HDL-C is much less than CRP, ESR, and proinflammatory cytokines expense, it is reasonable to suggest HDL-C as a novel biomarker for patients' inflammation evaluation with high clinical and financial performance.

The associations between low levels of HDL-C and severe inflammation in IBD supports the notion that HDL-C may represent a therapeutic target for inflammation (64). Several mechanisms are involved in its anti-inflammatory property. ApoAI mediates removal of membrane cholesterol and may affect antigen presentation and the induction of adaptive immune response (65). In dependent of HDL cholesterol efflux capacity, HDL also shown anti-inflammatory capacity by suppressing TNFα-induced vascular cell adhesion molecule-1 mRNA expression in endothelial cells (66). Furthermore, the antioxidant property of HDL-C reduces free radicals and oxidative stress which have been implicated in the pathogenesis of IBD (61). In endothelial cells, HDL-C inhibits inflammation by reducing activation of NF-κB and by inhibiting inflammasome activation (67). In macrophages, HDL-C sequesters lipopolysaccharide (68), suppress TLR4 expression on cell surface (69), and also blocks a broad spectrum of TLR-mediated inflammatory responses in an ATF3-dependent manner (36). Our study and the work of others provide evidence for HDL-C playing an important role in modulating the macrophage immune response in non-CV diseases like IBD (70, 71). By using a third generation of CETPi, which had a high selection on increasing HDL-C, we found that macrophage activation and polarization were influenced by evacetrapib in DSS-induced colitis. Given that the DSS colitis model is primarily driven by macrophages, we evaluated evacetrapib in a T-cell–dominated IL-10 knockout mouse model. Preventive effects of evacetrapib were absent in this model, further indicating that evacetrapib's mechanism of action likely involves macrophages (S Fig. 7). Macrophages in the gut promote stem cell differentiation and play critical role in maintaining immune homeostasis and barrier functions, but also affect the occurrence and development of diseases by TNFα-mediated proinflammatory circuit (61, 72). ATF3 is a transcriptional repressor for proinflammatory cytokines including IL-6, IL-12p40, and cholesterol 25-hydroxylase (Ch25h) (73) and its expression is essential for HDL-C mediates anti-inflammatory transcriptional reprogramming of macrophages (36). In our mouse model, *Atf3* expression in colon was increased by evacetrapib treatment but decreased in CETP-Tg mice, suggesting HDL-C improves IBD severity via inducing ATF3 and promoting an anti-inflammatory macrophages reprogramming.

CETP is a 74-KDa plasma glycoprotein that mediates the bidirectional movement of cholesteryl esters from HDL-C particles to TG-rich lipoproteins like VLDL (74). This exchange depletes HDL-C particles of their cholesterol content, induces HDL catabolism, and reduces the plasma concentration of HDL-C. Pharmacological inhibitors of CETP exist, from the perspective of clinical translation, contemporary CETPi are attractive in that they increase HDL-C via a mechanism relevant to the metabolic changes that occur during IBD, and have been shown to be safe and well tolerated in large clinical trials of patients with CVD after several generation development, although torcetrapib (first generation drug of CETPi) was associated with increased risk of mortality from infection in humans (30, 31, 32, 33). Notably, increasing HDL-C level by CETPi had no benefits for CVD but its anti-inflammatory property may ameliorate sepsis-induced mortality (35). Intriguingly, CETPi therapy could also reduce the incidence and progression of diabetes (75). In our study, CETP was inhibited for a subchronic or acute time span, which mimics what could occur in a clinical trial in humans. In different intervention settings, CETPi showed constant effect on improving experimental colitis outcome. Therefore, on the basis of previous literature and the experimental observations from this study, the CETPi appears to be effective to control inflammation in IBD and even other autoimmune and chronic inflammatory diseases. Although these specific therapies have not translated to clinical use, CETPi shows a promising future in non-CV diseases.

CETP is present in a variety of species like monkey and rabbit while mice and rats had low expression and activity (76). Trinder *et al*. also found anacetrapib, another third generation CETPi, shown no effects on sepsis survival in non-CETP-Tg mice (35). To our surprise, evacetrapib did elevate HDL-C level in WT mice and protected those mice from chemically induced colitis. Elevated HDL-C and HDL-C/LDL-C ratio were consistently observed in mice serum lipids profile under both chow diet and HFD feeding among WT mice; however, LDL-C or TG level was not influenced by evacetrapib treatment, so it is reasonable to think the therapy effect is dependent on changes to HDL-C. Although we cannot rule out the direct effects of evacetrapib or the indirect effects of evacetrapib increased HDL-C in our study, we found enhanced gut barrier function in evacetrapib treatment, which may also play a critical role in the protection of injury-induced colitis. This protective effect of evacetrapib is important especially for patients who had mutation in *CETP* genes and had low expression and activity. At the same time, the protective effects of evacetrapib may also be interesting to explore in other autoimmune and inflammatory disease model among WT mice to get a better understanding.

We observed that CETP inhibitor (CETPi) with evacetrapib more effectively increased HDL levels and ameliorated colitis in the context of an HFD. Research indicates that CETP protein levels rise in mice on an HFD, with activity increasing by 192% and HDL levels significantly decreasing (77). Consequently, evacetrapib's inhibition of CETP results in elevated HDL levels. Similarly, clinical trials have shown more pronounced HDL increases in patients with HFDs, though evacetrapib did not significantly reduce cardiovascular event risk (78).

Several limitations need to be considered. First, we proved the benefits of CETPi on experimental colitis in mice, but whether use of CETPi to increase HDL-C during human IBD will improve clinical outcomes requires further study in randomized controlled trials. Second, the relationship of HDL-C and risk of infectious disease occurred in a U-shaped manner (42), suggesting a window of ideal HDL-C levels is needed to maintain health. So, the preferred HDL-C goal in IBD patients is worth further exploring. Last, but not least, inflammation affects HDL-C function (71) and may convert HDL-C from anti-inflammatory to proinflammatory functions (70). So, not only the quantity of total HDL-C concentration but also the quality of HDL-C particles in IBD should be studied in future.

In sum, the current work provided evidence confirming that HDL-C levels correlate with disease activity in human autoimmune subjects. Moreover, we show that lower HDL-C level is associated with severe inflammation among individuals with IBD, predicts future disease activity and that HDL-C levels increase with successful biologic therapy. Moreover, subchronic and acute pharmacological inhibition of CETP improved colitis outcome in a mouse model and was associated with changes in inflammatory macrophage markers and gut barrier function.

## Data availability

All data needed to evaluate the conclusions in the paper are present in the paper or the supplemental data.

## Supplemental data

This article contains supplemental data.

## Conflict of interests

The authors declare that they have no conflicts of interest with the contents of this article.
