# Supplementary material for: Targeting to high density lipoprotein cholesterol: new insights for inflammatory bowel disease treatment
Source: J Lipid Res. 2025 Jun 6;66(7):100836. doi: 10.1016/j.jlr.2025.100836 (PMC12273436; doi:10.1016/j.jlr.2025.100836)
Supplement: Supplementary Material [file mmc1.docx]

**Supplemental Materials**

**List of Supplementary Materials**

S Fig1 Forest plot of serum lipids profile among CD patients, related to Table 1

S Fig2 Forest plot of serum lipids profile among UC patients, related to Table 1

S Fig3 HDL-C cut-off based on CRP level, related to Table 4

S Fig4 Lipid particle size in serum among CD and UC patients

S Fig5 Signature genes expression, related to Figure 6

S Fig6 Signature genes expression, related to Figure 9

S Fig7 Evacetrapib did not relieve colitis in the IL10 knockout model

S Table 1 Nutritional and inflammatory markers of different Montreal Classification among CD patients

S Table 2 Univariate and multivariate regression analyses between clinical variables and CDAI in CD patients or CRP in UC patients.

S Table 3 RT qPCR primers sequence

**S Fig1 Forest plot of serum lipids profile among CD patients, related to Table 1**

(A) Serum triglyceride (TG) level.

(B) Serum total cholesterol (TC) level.

(C) Serum low density lipoprotein cholesterol (LDL-C) level.

(D) Serum high density lipoprotein cholesterol (HDL-C) level.

**S Fig2 Forest plot of serum lipids profile among UC patients, related to Table 1**

(A) Serum triglyceride (TG) level.

(B) Serum total cholesterol (TC) level.

(C) Serum low density lipoprotein cholesterol (LDL-C) level.

(D) Serum high density lipoprotein cholesterol (HDL-C) level.

**S Fig3 HDL-C cut-off based on CRP level, related to Table 2**

(A) Serum lipids grouped by CRP Level.

(B) & (C) ROC curve of HDL-C level by CRP normal vs abnormal.


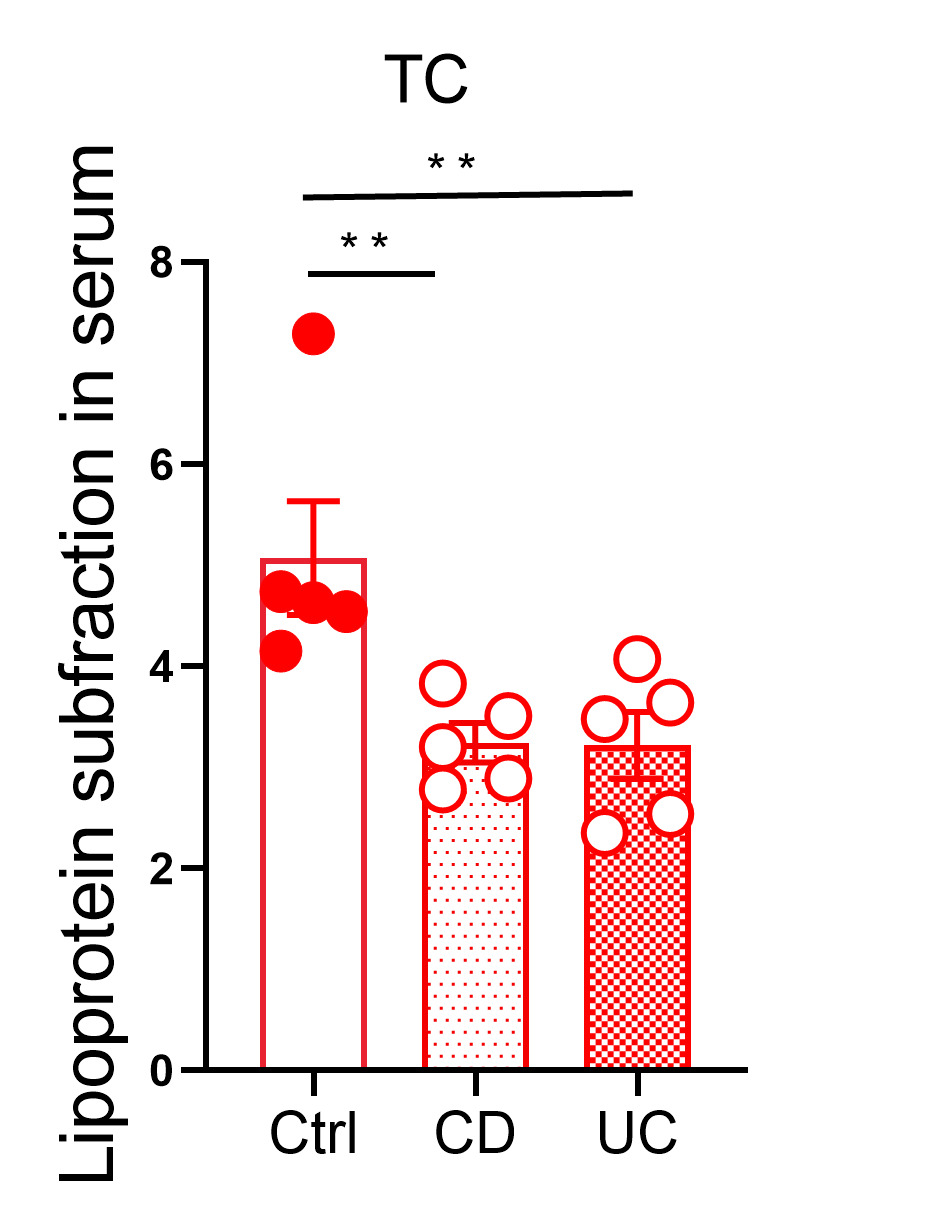

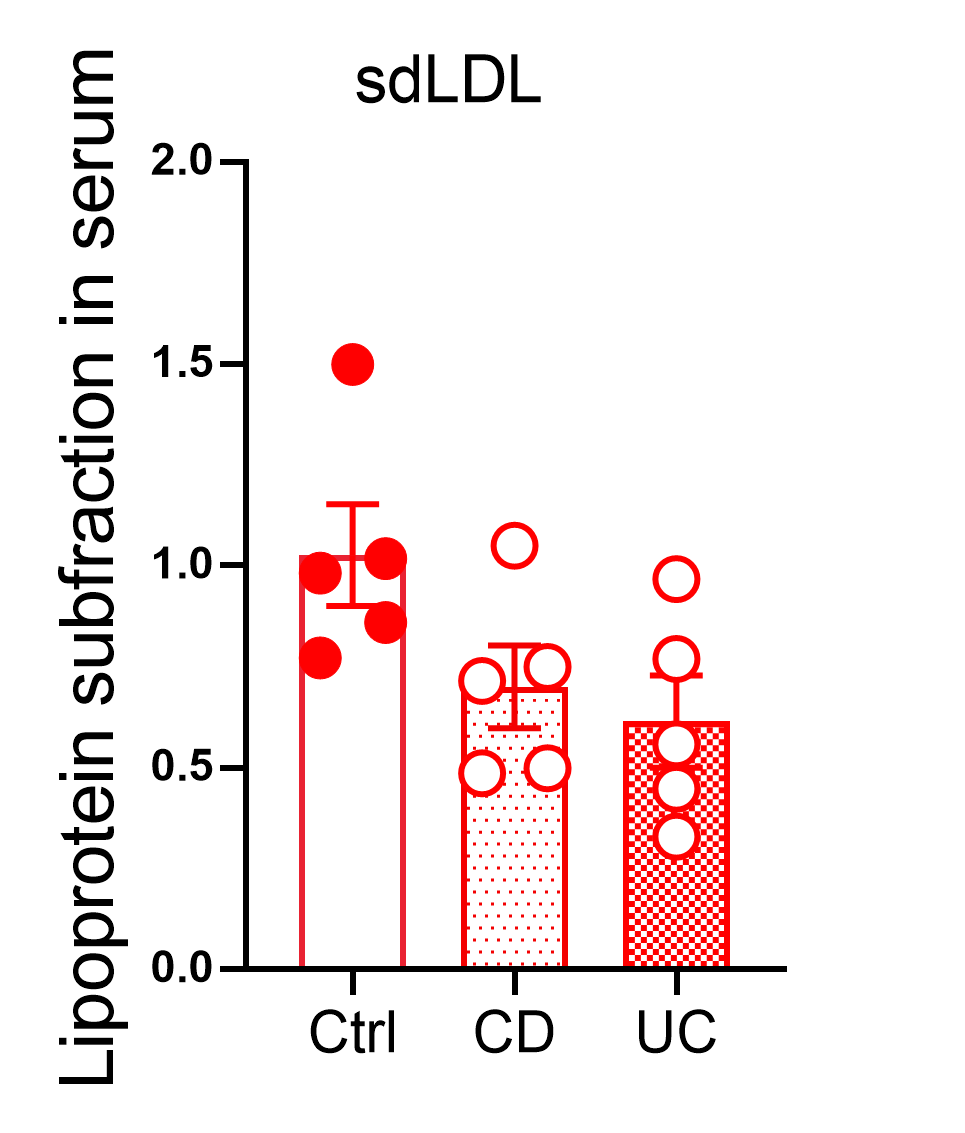

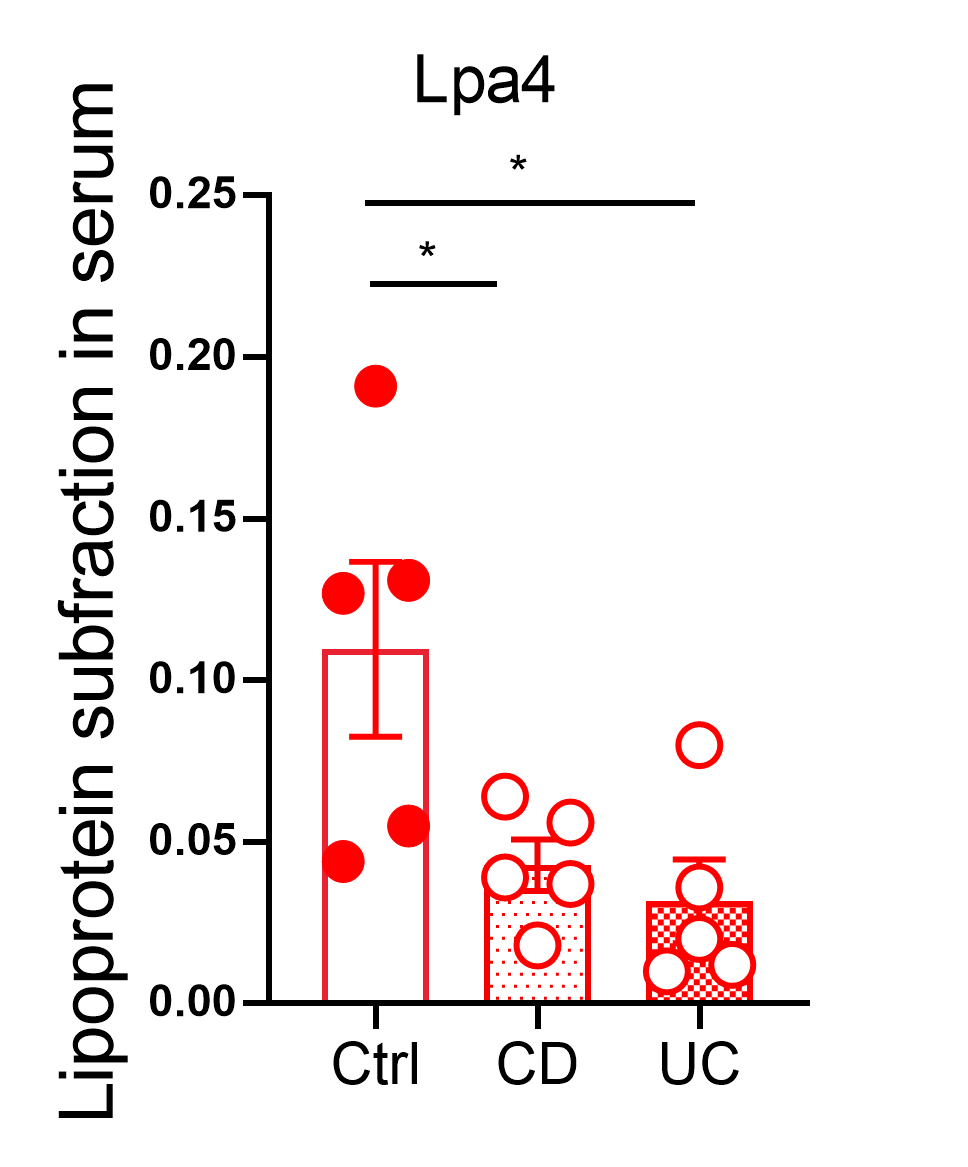

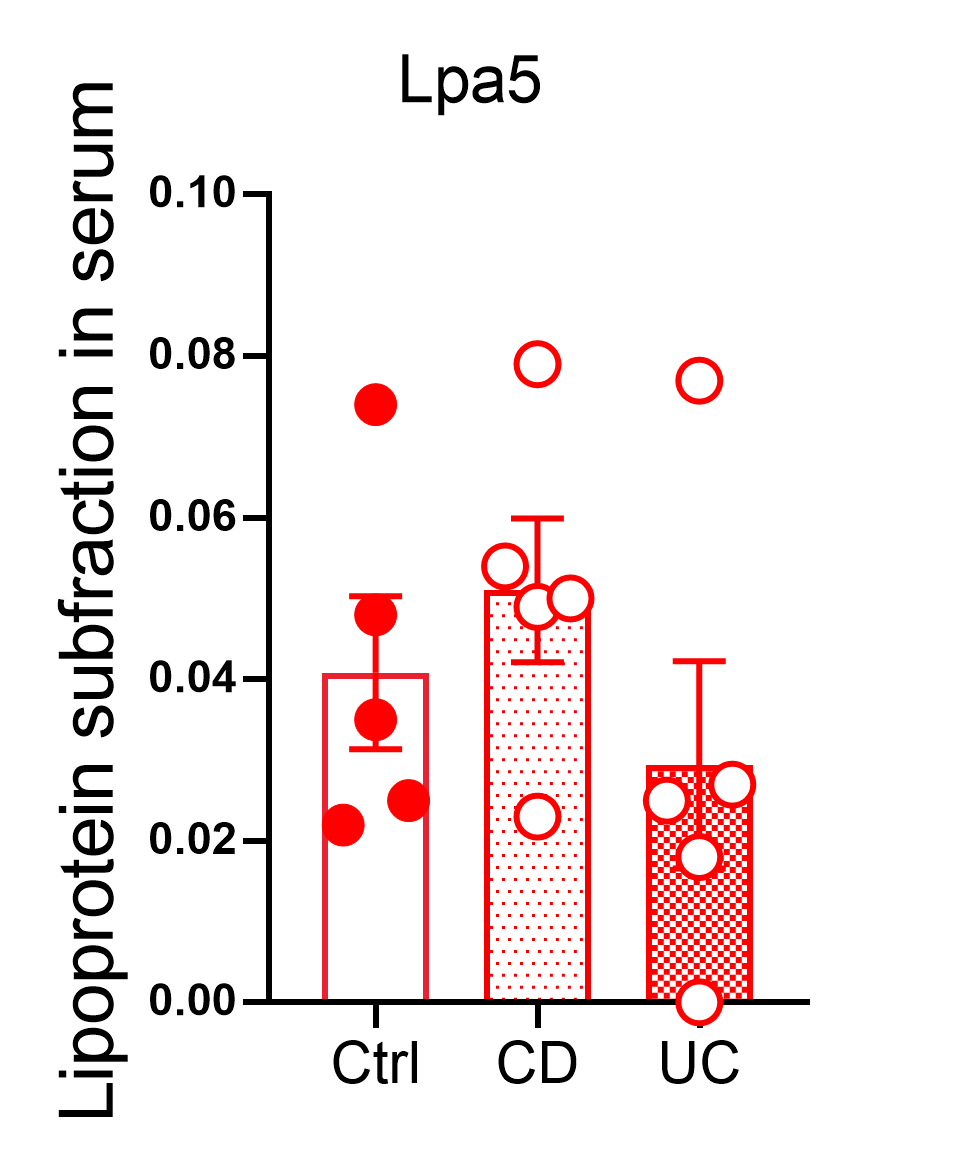

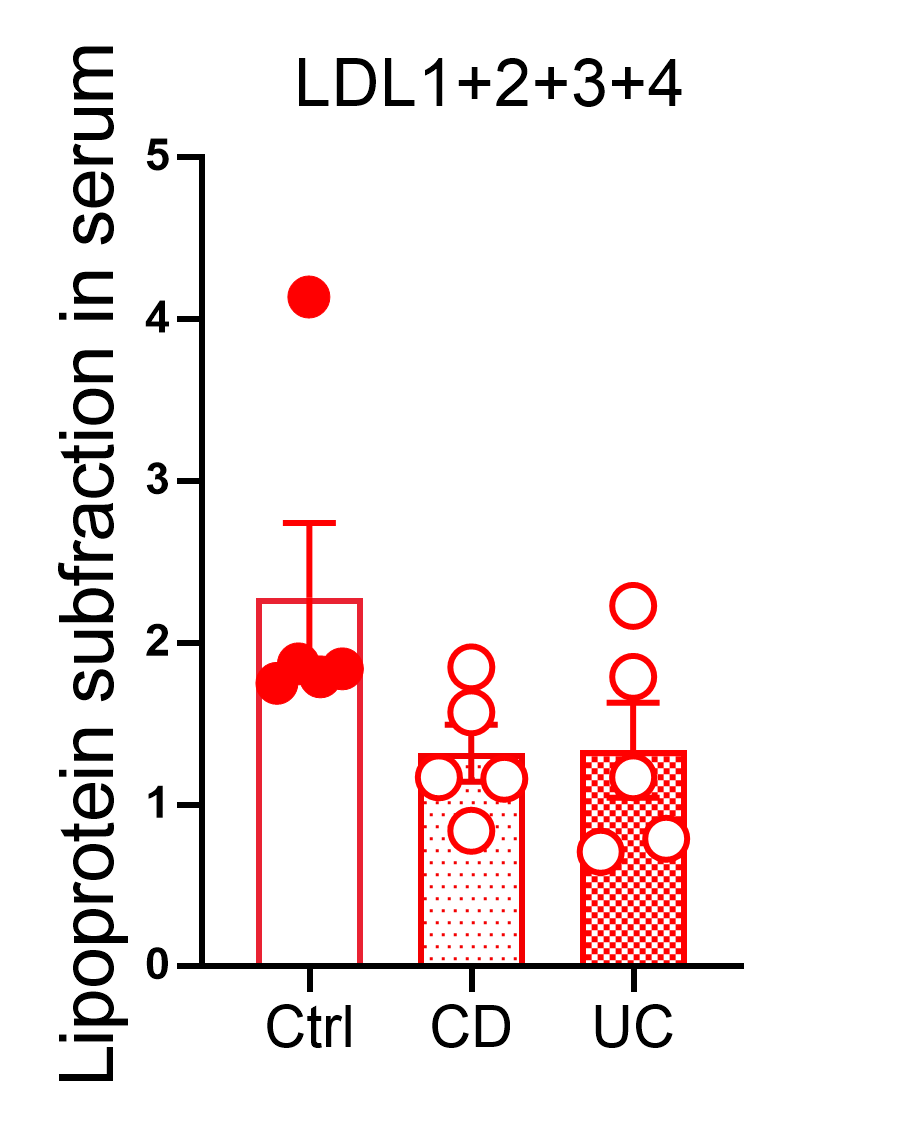


**S Fig4 Lipid particle size in serum among representative CD and UC patients**

Lipid particle size in serum were analyzed among 5 CD patients, 5 UC patients and 5 healthy controls. The patients were matched according to BMI, total protein level and other nutritional status indicators.

**S Fig5 Signature genes expression, related to Figure 6**

WT male mice were feed on chow diet supplied with Evacetrapib in drinking water and outcomes were analyzed before or after DSS treatment. Expression of indicated genes in distal colon tissue, normalized to *Gapdh*.

**S Fig6 Signature genes expression, related to Figure 9**

WT male mice were feed on chow diet and got Evacetrapib by *i.p*. injection every other day and outcomes were analyzed after *C. rodentium* infection. Expression of indicated genes in distal colon tissue, normalized to *Gapdh*.

**
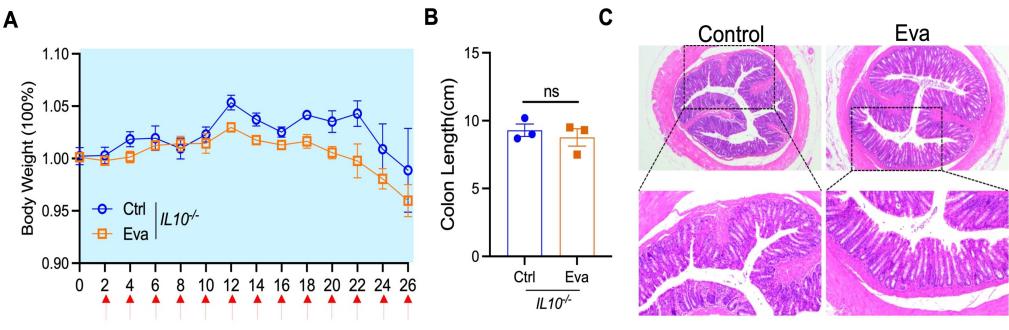
**

**S Fig7 Evacetrapib did not relieve colitis in the IL10 knockout model**

(A) Body weights shown as percentage of starting weight (n = 3 per group).

(B) Colon length.

(C) Representative images of H&E of tissue sections from distal colon.

**S Table 1 Nutritional and inflammatory markers of different Montreal Classification among CD patients**

|  | **Nutritional condition** | | |  | **Serum lipids** | | | |  | **y** | **inflammatory markers** | | | | |  |
| --- | --- | --- | --- | --- | --- | --- | --- | --- | --- | --- | --- | --- | --- | --- | --- | --- |
|  | **BMI** | **ALB** | **TP** |  | **HDL-C** | **LDL-C** | **TG** | **TC** | |  | **CRP** | **Fibrinogen** | **WBC** | **ESR** | **HBI** | |
| **Age** |  |  |  |  |  |  |  |  | |  |  |  |  |  |  | |
| A1（n=8） | 0.4523^a^ | 0.7001^a^ | 0.2126^a^ |  | 0.9421^a^ | 0.9914^a^ | 0.9954^a^ | 0.9998^a^ | |  | 0.9827^a^ | 0.9862^a^ | 0.9027^a^ | 0.9827^a^ | 0.5474^a^ | |
| A2（n=60） | **<0.000**^b^ | 0.8589^b^ | 0.8293^b^ |  | 0.6614^b^ | **0.0021**^b^ | **0.0006**^b^ | **0.0015**^b^ | |  | 0.9622^b^ | 0.8570^b^ | 0.9076^b^ | 0.9950^b^ | 0.9719^b^ | |
| A3（n=27） | **<0.0001**^c^ | 0.8940^c^ | 0.1386^c^ |  | 0.9798^c^ | 0.0900^c^ | 0.0588^c^ | 0.1064^c^ | |  | 0.7411^c^ | 0.8930^c^ | 0.7958^c^ | 0.9931^c^ | 0.5078^c^ | |
| **Loacation** |  |  |  |  |  |  |  |  | |  |  |  |  |  |  | |
| L1（n=33） | 0.0500^a^ | 0.4703^a^ | 0.8285^a^ |  | 0.5354^a^ | 0.9995^a^ | 0.3723^a^ | 0.9989^a^ | |  | 0.9249^a^ | 0.8975^a^ | 0.9780^a^ | 0.9885^a^ | 0.7308^a^ | |
| L2（n=6） | 0.1172^b^ | 0.3910^b^ | 0.8898^b^ |  | 0.6058^b^ | 0.9316^b^ | 0.4665^b^ | 0.9261^b^ | |  | 0.9859^b^ | 0.6523^b^ | 0.8890^b^ | 0.7167^b^ | 0.8412^b^ | |
| L3（n=53） | 0.6551^c^ | 0.9785^c^ | 0.1029^c^ |  | 0.9573^c^ | 0.8001^c^ | 0.9149^c^ | 0.6939^c^ | |  | 0.5381^c^ | 0.6865^c^ | 0.3990^c^ | 0.1744^c^ | 0.9069^c^ | |
| **Behavior** |  |  |  |  |  |  |  |  | |  |  |  |  |  |  | |
| B1（n=41） | 0.3167^a^ | 0.6416^a^ | **0.0038**^a^ |  | 0.5923^a^ | 0.9684^a^ | 0.3304^a^ | 0.8346^a^ | |  | 0.5883^a^ | 0.4977^a^ | 0.7043^a^ | 0.1495^a^ | 0.3191^a^ | |
| B2（n=38） | 0.3609^b^ | 0.5996^b^ | **0.0010**^b^ |  | 0.9926^b^ | 0.3211^b^ | ＞0.9999^b^ | 0.5679^b^ | |  | 0.3044^b^ | 0.0794^b^ | 0.9609^b^ | **0.0498**^b^ | 0.9943^b^ | |
| B3（n=5） | 0.8152^c^ | 0.6333^c^ | **0.0221**^c^ |  | 0.9932^c^ | 0.4533^c^ | 0.2211^c^ | 0.5541^c^ | |  | 0.1847^c^ | 0.1646^c^ | 0.8576^c^ | 0.2109^c^ | 0.8598^c^ | |

p value. Bold values are for p<0.05. ^a^Comparison between Group1 and Group2; ^b^Comparison between Group2 and Group3; ^c^Comparison between Group1 and Group3; CD:Crohn’s disease; n,number of patients per group; A1:≦16;A2:17-40; A3:＞40; L1:Ileal; L2:Colonic; L3:Ileocolonic; B1:Non-stricturing, Non- penetrating; B2:Stricturing; B3:Penetrating; BMI:Body Mass Index; ALB:Serum albumin; TP:Total protein; HDL-C:High-density lipoprotein cholesterol; LDL-C: Low-density lipoprotein cholesterol; TG:Triglycerides; TC:Total cholesterol; CRP:C-reactive protein; WBC:White Blood Cells; ESR:Erythrocyte Sedi mentation Rate; HBI:Harvey Bradshaw.

**S Table 2 Univariate and multivariate regression analyses between clinical variables** **and CDAI in CD patients or CRP in UC patients.**

| Risk factor | | Binary logistic regression | | |  | Multiple logistic regression | | |
| --- | --- | --- | --- | --- | --- | --- | --- | --- |
|  |  | Odds ratio | 95%CI | p value |  | Odds ratio | 95%CI | p value |
| CD | Age | 1.022 | 0.986-1.058 | 0.240 |  |  |  |  |
|  | Gender | 2.008 | 0.740-5.449 | 0.171 |  |  |  |  |
|  | BMI | 0.760 | 0.627-0.922 | 0.005* |  | 0.777 | 0.574-1.050 | 0.100 |
|  | Montreal classification L | 1.315 | 0.865-1.999 | 0.201 |  |  |  |  |
|  | Montreal classification B | 1.350 | 0.751-2.426 | 0.317 |  |  |  |  |
|  | Montreal classification P | 0.930 | 0.406-2.132 | 0.864 |  |  |  |  |
|  | TG | 0.666 | 0.341-1.301 | 0.234 |  |  |  |  |
|  | TC | 0.509 | 0.304-0.851 | 0.010* |  | 0.749 | 0.020-28.144 | 0.876 |
|  | HDL-C | 0.002 | 0.001-0.048 | <0.001* |  | <0.001 | 0.000-0.093 | 0.004* |
|  | LDL-C | 0.450 | 0.214-0.946 | 0.035* |  | 0.926 | 0.009-100.283 | 0.974 |
|  | TP | 0.920 | 0.867-0.977 | 0.007* |  | 0.958 | 0.835-1.099 | 0.544 |
|  | ALB | 0.782 | 0.699-0.874 | <0.001* |  | 1.001 | 0.774-1.294 | 0.995 |
|  | WBC | 1.122 | 0.967-1.302 | 0.128 |  |  |  |  |
|  | Fibrinogen | 2.968 | 1.759-5.008 | <0.001* |  | 2.266 | 0.931-5.513 | 0.072 |
| UC | Age | 0.997 | 0.969-1.027 | 0.860 |  |  |  |  |
|  | Gender | 1.309 | 0.534-3.209 | 0.556 |  |  |  |  |
|  | TG | 1.119 | 0.760-1.649 | 0.569 |  |  |  |  |
|  | TC | 0.672 | 0.433-1.042 | 0.075 |  |  |  |  |
|  | HDL-C | 0.024 | 0.003-0.194 | <0.001* |  | 0.016 | 0.001-0.262 | 0.004* |
|  | LDL-C | 0.429 | 0.208-0.883 | 0.022* |  | 1.201 | 0.463-3.110 | 0.707 |
|  | TP | 0.910 | 0.847-0.979 | 0.011* |  | 0.956 | 0.876-1.044 | 0.317 |
|  | ALB | 0.969 | 0.938-1.002 | 0.065 |  |  |  |  |
|  | WBC | 1.476 | 1.176-1.853 | <0.001* |  | 1.361 | 1.067-1.736 | 0.013* |
|  | Fibrinogen | 1.037 | 0.918-1.173 | 0.557 |  |  |  |  |

p value. Bold values are for p<0.05. L:location;L1:Ileal; L2:Colonic; L3:Ileocolonic; B:behaviour; B1:Non-stricturing, Non- penetrating; B2:Stricturing; B3:Penetrating; P:Perianal lesions; BMI:Body Mass Index; ALB:Serum albumin; TP:Total protein; HDL-C:High-density lipoprotein cholesterol; LDL-C: Low-density lipoprotein cholesterol; TG:Triglycerides; TC:Total cholesterol; CRP:C-reactive protein; WBC:White Blood Cells;

**S Table 3 RT qPCR primers sequence**
